# Supplementary material for: Prospective Evaluation over 15 Years of Six Breast Cancer Risk Models
Source: Cancers (Basel). 2021 Oct 16;13(20):5194. doi: 10.3390/cancers13205194 (PMC8534072; doi:10.3390/cancers13205194)
Supplement: Supplementary file 1 [file cancers-13-05194-s001.zip › MCCS_Questionnaire_BASELINE.pdf]

ENGLISH LIFESTYLE QUESTIONNAIRE

FIRSTLY SOME QUESTIONS ABOUT YOUR SOCIAL LIFE

1. Including yourself, how many people live in your household?

- ❑ 1
- ❑ 2
- ❑ 3 or 4
- ❑ 5 or more

2. Apart from those who live with you, how many relatives do you usually see at least once a month?

- ❑ none
- ❑ 1 or 2
- ❑ 3 to 4
- ❑ 5 to 9
- ❑ 10 or more

3. Excluding your relatives, how many friends do you have who you could visit at any time without an invitation?

- ❑ none
- ❑ 1 or 2
- ❑ 3 to 4
- ❑ 5 to 9
- ❑ 10 or more

4. How many hours a week, if any, do you spend involved in social activities outside your home or work? *(e.g. ethnic clubs, work clubs, church groups or sporting groups and other community groups or regular social meetings with friends)*?

- ❑ none
- ❑ 1 or 2
- ❑ 3 to 4
- ❑ 5 to 9
- ❑ 10 or more

NOW I WOULD LIKE TO ASK YOU SOME QUESTIONS ABOUT YOUR BACKGROUND

5. What is the highest level of education you have completed?

- ❑ never attended school
- ❑ some primary school
- ❑ completed primary school
- ❑ some high/technical school
- ❑ completed high/technical school
- ❑ other qualification *(eg. trade certificate)*
- ❑ some study towards a tertiary degree or diploma
- ❑ completed tertiary degree or diploma

6. What is/was your usual occupation?

NOW I WOULD LIKE TO ASK SOME QUESTIONS ABOUT YOUR FAMILY AND THEIR MEDICAL HISTORY

7. Is your father living?

   ❑ yes ❑ no

8. How old is/was your father?

9. Is your mother living?

   ❑ yes ❑ no

10. How old is/was your mother?

11. Have any of these relatives had:

	Father	Mother	Brother	Sister	Son	Daughter
Heart Attack	❑	❑	❑	❑	❑	❑
Cancer	❑	❑	❑	❑	❑	❑
Diabetes	❑	❑	❑	❑	❑	❑
Stroke	❑	❑	❑	❑	❑	❑

12. How many brothers do/did you have?

13. How many sisters do/did you have?

14. How many children do you have?

15. Are you a twin?

    ❑ yes ❑ no

NOW SOME QUESTIONS ABOUT YOUR HEALTH

16. What was your weight when you were between 18 and 21 years of age?

17. What is the heaviest weight you have been? *(exclude pregnancy weight)*

18. At what age did you achieve your heaviest weight?

19. Over the last five years has your weight:

    ❑ increased by more than 5 kg (11 lbs)
    ❑ decreased by more than 5 kg (11 lbs)
    ❑ stayed much the same
    ❑ don't know

20. Has the doctor ever told you that you have had:

	Yes	No	If yes, how old were you?
Asthma or wheezy breathing	❑	❑	....
If yes, do you take medications	❑	❑	....
Angina	❑	❑	....
If yes, do you take medications	❑	❑	....
Hypertension *(high blood pressure)*	❑	❑	....
If yes, do you take tablets	❑	❑	
Diabetes mellitus *(sugar diabetes)*	❑	❑	....
If yes, do you take insulin injections	❑	❑	....
do you take tablets	❑	❑	
Arthritis or rheumatism	❑	❑	....
If yes, do you take aspirin?	❑	❑	
Cancer	❑	❑	....
If yes, what type of cancer			....
Kidney stones	❑	❑	....
Gallstones	❑	❑	....
If yes, have you had your gallbladder removed	❑	❑	
Heart attack	❑	❑	....
Stroke	❑	❑	....

NOW SOME QUESTIONS ABOUT YOUR HABITS THAT MAY AFFECT YOUR HEALTH.

MOST PEOPLE VARY IN THEIR HABITS THROUGH LIFE, I WOULD LIKE TO NOW ASK ABOUT YOUR CURRENT HABITS AND THEN GO BACKWARDS IN TIME ASKING YOU ABOUT YOUR HABITS AT VARIOUS AGES

21. Have you ever smoked at least 7 cigarettes a week for at least a year?

❑ yes ❑ no

☛ *If NO, go to Q 29*

22. How old were you when you first started smoking at least 7 cigarettes a week?

23. How many cigarettes do/did you smoke a day on average?

24. Do you now smoke at least 7 cigarettes a week?

❑ yes ❑ no

25. If NO, at what age did you stop smoking at least 7 cigarettes a week?

26. Between the age you started and the age you stopped *(or now for current smokers)* have you not smoked for periods of at least a year?

❑ yes ❑ no

27. If YES, for how many years altogether did you not smoke during this period?

28. So altogether you have smoked cigarettes for how many years?

29. Have you ever smoked at least seven cigars a week for at least at year?

❑ yes ❑ no

30. Have you ever smoked at least seven pipes of tobacco a week for at least a year?

❑ yes ❑ no

31. Have you ever drunk at least 12 alcoholic drinks in a year? *(sips and tastes don't count)*

❑ yes ❑ no

If NO, go to Q 86

32. How old were you when you first drank at least 12 alcoholic drinks in a year? *(sips and tastes don't count)*

33. Between the ages of 10-14 did you drink beer ❑ yes ❑ no, wine ❑ yes ❑ no or drinks containing spirits ❑ yes ❑ no?

(IF NO, to all three, go to Q41)

34. How often did you drink beer?

. . . . . ❑ day ❑ week ❑ month ❑ year

35. When you drank beer how much did you drink on average?

. . . . . ❑ glasses ❑ pots ❑ cans ❑ stubbies ❑ bottles

36. How often did you drink wine?

. . . . . ❑ day ❑ week ❑ month ❑ year

37. When you drank wine how much did you drink on average?

. . . . . ❑ glasses ❑ bottles ❑ litres

38. How often did you drink drinks containing spirits?

. . . . . ❑ day ❑ week ❑ month ❑ year

39. When you drank drinks containing spirits, how many did you drink on average?

. . . . . ❑ glasses

40. Between the ages of 10-14, what was the most alcoholic drinks that you consumed on any one occasion?

. . . .

41. Between the ages of 15-19 did you drink beer ❑ yes ❑ no, wine ❑ yes ❑ no or drinks containing spirits ❑ yes ❑ no?

(IF NO, to all three, go to Q49)

42. How often did you drink beer?

. . . . . ❑ day ❑ week ❑ month ❑ year

43. When you drank beer how much did you drink on average?

. . . . . ❑ glasses ❑ pots ❑ cans ❑ stubbies ❑ bottles

44. How often did you drink wine?

. . . . . ❑ day ❑ week ❑ month ❑ year

45. When you drank wine how much did you drink on average?

. . . . . ❑ glasses ❑ bottles ❑ litres

46. How often did you drink drinks containing spirits?

. . . . . ❑ day ❑ week ❑ month ❑ year

47. When you drank drinks containing spirits, how many did you drink on average?

. . . . . ❑ glasses

48. Between the ages of 15-19, what was the most alcoholic drinks that you consumed on any one occasion?

. . . .

49. Between the ages of 20-29 did you drink beer ❑ yes ❑ no, wine ❑ yes ❑ no or drinks containing spirits ❑ yes ❑ no?

(IF NO, to all three, go to Q56)

50. How often did you drink beer?

. . . . . ❑ day ❑ week ❑ month ❑ year

51. When you drank beer how much did you drink on average?

. . . . . ❑ glasses ❑ pots ❑ cans ❑ stubbies ❑ bottles

52. How often did you drink wine?

. . . . . ❑ day ❑ week ❑ month ❑ year

53. When you drank wine how much did you drink on average?

. . . . . ❑ glasses ❑ bottles ❑ litres

54. How often did you drink drinks containing spirits?

. . . . . ❑ day ❑ week ❑ month ❑ year

55. When you drank drinks containing spirits, how many did you drink on average?

. . . . . ❑ glasses

56. Between the ages of 30-39 did you drink beer ❑ yes ❑ no, wine ❑ yes ❑ no or drinks containing spirits ❑ yes ❑ no?

(IF NO, to all three, go to Q63)

57. How often did you drink beer?

. . . . . ❑ day ❑ week ❑ month ❑ year

58. When you drank beer how much did you drink on average?

. . . . . ❑ glasses ❑ pots ❑ cans ❑ stubbies ❑ bottles

59. How often did you drink wine?

. . . . . ❑ day ❑ week ❑ month ❑ year

60. When you drank wine how much did you drink on average?

. . . . . ❑ glasses ❑ bottles ❑ litres

61. How often did you drink drinks containing spirits?

. . . . . ❑ day ❑ week ❑ month ❑ year

62. When you drank drinks containing spirits, how many did you drink on average?

. . . . . ❑ glasses

63. Between the ages of 40-49 did you drink beer ❑ yes ❑ no, wine ❑ yes ❑ no or drinks containing spirits ❑ yes ❑ no?

(IF NO, to all three, go to Q70)

64. How often did you drink beer?

. . . . . ❑ day ❑ week ❑ month ❑ year

65. When you drank beer how much did you drink on average?

. . . . . ❑ glasses ❑ pots ❑ cans ❑ stubbies ❑ bottles

66. How often did you drink wine?

. . . . . ❑ day ❑ week ❑ month ❑ year

67. When you drank wine how much did you drink on average?

. . . . . ❑ glasses ❑ bottles ❑ litres

68. How often did you drink drinks containing spirits?

. . . . . ❑ day ❑ week ❑ month ❑ year

69. When you drank drinks containing spirits, how many did you drink on average?

. . . . . ❑ glasses

70. Between the ages of 50-59 did you drink beer ❑ yes ❑ no, wine ❑ yes ❑ no or drinks containing spirits ❑ yes ❑ no?

(IF NO, to all three, go to Q77)

71. How often did you drink beer?

. . . . . ❑ day ❑ week ❑ month ❑ year

72. When you drank beer how much did you drink on average?

. . . . . ❑ glasses ❑ pots ❑ cans ❑ stubbies ❑ bottles

73. How often did you drink wine?

. . . . . ❑ day ❑ week ❑ month ❑ year

74. When you drank wine how much did you drink on average?

. . . . . ❑ glasses ❑ bottles ❑ litres

75. How often did you drink drinks containing spirits?

. . . . . ❑ day ❑ week ❑ month ❑ year

76. When you drank drinks containing spirits, how many did you drink on average?

. . . . . ❑ glasses

77. Between the ages of 60-69 did you drink beer ❑ yes ❑ no, wine ❑ yes ❑ no or drinks containing spirits ❑ yes ❑ no?

(IF NO, to all three, go to Q84)

78. How often did you drink beer?

. . . . . ❑ day ❑ week ❑ month ❑ year

79. When you drank beer how much did you drink on average?

. . . . . ❑ glasses ❑ pots ❑ cans ❑ stubbies ❑ bottles

80. How often did you drink wine?

. . . . . ❑ day ❑ week ❑ month ❑ year

81. When you drank wine how much did you drink on average?

. . . . . ❑ glasses ❑ bottles ❑ litres

82. How often did you drink drinks containing spirits?

..... ❑ day ❑ week ❑ month ❑ year

83. When you drank drinks containing spirits, how many did you drink on average?

..... ❑ glasses

84. What alcoholic beverages, if any, did you drink on each day during the last week?

MONDAY ....
TUESDAY ....
WEDNESDAY ....
THURSDAY ....
FRIDAY ....
SATURDAY ....
SUNDAY ....

85. Is the amount you drank last week more, less or about the same as you would drink most weeks?

NOW WE WOULD LIKE TO ASK YOU SOME QUESTIONS RELATING TO PHYSICAL ACTIVITY

87. ~~86.~~ On average, *(e.g., over the last 6 months)* how many times a week do you exercise vigorously for a period of at least 20 minutes? *("**Vigorously**" means making you sweat or feel out of breath, and includes such activities as swimming, tennis, netball, athletics and running.)*

❑ none at all —— Go to Q89
❑ once or twice a week
❑ three or more time a week

88. ~~87.~~ If you exercise vigorously *"3 or more times a week"*, for how long have you been doing this level of activity?

❑ less than 3 months
❑ more than 3 month but less than 1 year
❑ more than 1 year but less than 5 years
❑ 5 years or more

89. ~~88.~~ On average, *(e.g. over the last 6 months)* how many times a week do you engage in less vigorous exercise for recreation, sport or health and fitness purposes, which did not make you sweat or feel out of breath *(and includes such activities as bike riding, dancing, etc.)*

❑ not at all
❑ once or twice a week
❑ three or more times a week

86. ~~89.~~ On average, *(e.g., over the last 6 months)* how many times a week do you walk for recreation or exercise?

❑ not at all
❑ once or twice a week
❑ three or more times a week

90. **On average, in the course of your tasks at work and around the house, how much time are you involved in moderate to heavy physical exertion** ***(exertion which made you breathe harder or puff and pant).***

At Work

None at all ❐

Total time .... hours .... mins per ❐ day or ❐ week

At Home

None at all ❐

Total time .... hours .... mins per ❐ day or ❐ week

FOR WOMEN ONLY

91. How old were you when you had your first menstrual period? ....

92. Have you ever been pregnant? ❐ yes ❐ no

☞ **If NO, go to Q 94**

93. Please give details about all of your pregnancies irrespective of outcome *(include all miscarriages and terminations)*

Year pregnancy	for how many weeks were you pregnant?	live birth Yes/No	Breastfeeding ended (in months)

94. Have you ever used the contraceptive pill?

❐ no ❐ yes, in the past ❐ yes, currently

☞ **If NO, go to Q 97**

95. How old were you when you first started taking the contraceptive pill?
....

96. For how long have you used the pill altogether? ....

97. Have you had a menstrual period in the last 12 months?

❐ yes ❐ no

*** IF YES, STOP you have completed the questionnaire ***

98. If NO, how old were you when you had your last period? ....

99. Why have your periods stopped?

- ❑ naturally, as part of the change of life
- ❑ following a hysterectomy
- ❑ for another reason (specify) . . . .

100. Have you had a hysterectomy?

- ❑ yes
- ❑ no

☛ *If NO, go to Q 103*

101. How old were you when you had your hysterectomy?

102. Were your ovaries removed?

❑ yes ❑ no

103. Has a doctor prescribed female hormone medications such as oestrogen replacement pills or injections to reduce the symptoms of the menopause?

- ❑ no
- ❑ yes, in the past
- ❑ yes, currently

** *IF NO, STOP you have completed the questionnaire* **

104. How old were you when your first started taking female hormone or oestrogen replacement pills or injections?

105. How old were you when you last stopped taking female hormone or oestrogen replacement pills or injections?

106. When you were last taking female hormone or oestrogen replacement pills or injections do/did you regularly take any other pill for part of each month with them as part of your treatment?

❑ yes ❑ no

☛ *If NO, go to Q 108*

107. What was the name of the other pill that you take/took while taking female hormone or oestrogen replacement pills or injections?

108. For about how long altogether (have/did) you take(n) female hormone or replacement oestrogen pills or injections?
