# Supplementary material for: Prospective Evaluation over 15 Years of Six Breast Cancer Risk Models
Source: Cancers (Basel). 2021 Oct 16;13(20):5194. doi: 10.3390/cancers13205194 (PMC8534072; doi:10.3390/cancers13205194)
Supplement: Supplementary file 1 [file cancers-13-05194-s001.zip › MCCS_Questionnaire_FUP1.pdf]

HEALTH 2000

A study of lifestyle and health

FIRST FOLLOW-UP QUESTIONNAIRE

ID Number

Date of birth:

Please check the details above and correct any errors if necessary. If you are planning to move, write the new address (or a suitable postal address) in the space on the right.

Please read this page carefully before you start.

- Use a soft pencil only (preferrably a 2B pencil).
- Fill in the bubbles completely, as in the example ● **do not use** ✓ **or** ✗ .
- Only write in the space provided. If you need to give more information, use the yellow "extra comments" sheet.
- **Avoid leaving questions blank.** Try to answer every question, even if the answer is **no, never,** or **don't know.**
- Should you need any help in completing this questionnaire call: 9279 1323 (English), 9279 1322 (Greek) or 9279 1324 (Italian).
- **Do not fold this booklet.**
- When completed, check that you have answered all questions.
- We would be grateful if you could return this booklet and the yellow "extra comments" sheet in the envelope provided within a couple of weeks.
- **Do not staple the extra comments sheet to the booklet.**

OFFICE USE ONLY

ID NUMBER

0	0	0	0	0
1	1	1	1	1
2	2	2	2	2
3	3	3	3	3
4	4	4	4	4
	5	5	5	5
	6	6	6	6
	7	7	7	7
	8	8	8	8
	9	9	9	9

Thank you for your continuing contribution to Health 2000. We will contact you again in 3 to 4 years time.

02 03 04 05

A Quality Guaranteed Scannable Document for OpScan OMR/ICR Scanners. Printed in Australia by SCANFORM on © MarkScan **4372**

SECTION 1: HOSPITAL ADMISSIONS

1 **In the last 5 years, have you been admitted to hospital?** ○ No ○ Yes

If **YES,** fill in the details below (women include any maternity admissions)

Reason for hospital admission	Month / Year	Hospital

SECTION 2: FAMILY EVENTS

1 **What is your present marital status?**

○ never married ○ married / de facto ○ other committed relationship
○ separated ○ divorced ○ widowed

2 **Is your parent, brother, sister, child or spouse also in HEALTH 2000?** ○ No ○ Yes

If **YES,** fill out their name, their relationship to you, and (if known) their HEALTH 2000 Number.

Name	Relation to you	Health 2000 Number

3 **In the last 5 years, has a parent, brother, sister, child or spouse either been newly diagnosed with a serious illness (such as cancer) or died?** ○ No ○ Yes

If **YES,** please give the following details. **Note:** if a relative has died, give **Age** and **Date** of death as accurately as possible. If a relative is living give the **Age** and **Date** of diagnosis.

				DETAILS OF DIAGNOSIS OR DEATH		
**Name** (Optional)	**Relation to you**	**Living**	**Deceased**	**Illness / Cause of Death**	**Age**	**Date** (see note above)
		○	○			
		○	○			
		○	○			
		○	○			
		○	○			

SECTION 3: YOUR FAMILY DOCTOR OR LOCAL HEALTH CENTRE

As some people change their doctor from time to time, we would like to update our records in regard to the doctor or clinic you attend most often for your health care.

Please fill out the name and address of your current doctor or health centre.

NAME: ____________________

ADDRESS: ____________________

____________________ **POSTCODE:** __________

PHONE: ____________________

***** If not enough space, use yellow extra comments sheet *****

SECTION 4:

HEALTH CONDITIONS

Has a doctor *ever* told you that you have had the following?

Give an answer for each item. If **"Yes"**, give the year the diagnosis was made or the operation was performed.

EXAMPLE: If diagnosis of angina in 1995, enter

● Yes Year [9][5]
○ No 19
②③④⑤⑥⑦⑧●
⓪①②③④●⑥⑦⑧⑨

HEALTH CONDITION

Angina

○ Yes Year [ ][ ]
○ No 19

Heart by-pass surgery (Coronary artery by-pass graft)

○ Yes Year [ ][ ]
○ No 19

Heart angioplasty (balloon procedure)

○ Yes Year [ ][ ]
○ No 19

Heart attack (myocardial infarction, coronary occlusion)

○ Yes Year [ ][ ]
○ No 19

Heart failure (congestive cardiac failure, pulmonary oedema)

○ Yes Year [ ][ ]
○ No 19

Stroke

○ Yes Year [ ][ ]
○ No 19

High blood pressure (hypertension)

○ Yes Year [ ][ ]
○ No 19

High blood cholesterol

○ Yes Year [ ][ ]
○ No 19

Asthma (wheezy breathing)

○ Yes Year [ ][ ]
○ No 19

Diabetes

○ Yes Year [ ][ ]
○ No 19

Cancer (including lymphoma, leukaemia or melanoma)

○ Yes Year [ ][ ]
○ No 19

Type of cancer ____________________

Liver disease

○ Yes Year [ ][ ]
○ No 19

Type of liver disease ____________________

Gallstones

○ Yes Year [ ][ ]
○ No 19

Kidney stones

○ Yes Year [ ][ ]
○ No 19

Cataracts

○ Yes Year [ ][ ]
○ No 19

Fracture (broken bone(s))

○ Yes Year [ ][ ]
○ No 19

Type of fracture ____________________

Other severe or long term medical condition(s)

○ Yes Year [ ][ ]
○ No 19

Type of medical condition ____________________

○ Yes Year [ ][ ]
○ No 19

Type of medical condition ____________________

SECTION 5:

MEDICAL EXAMINATIONS

Have you had any of the following examinations or tests *in the last 5 years.*

Answer Yes or No for each item. If **Yes,** give the year in which you last had it performed.

Skin examination (doctor checks for skin cancer)

○ Yes Year [ ]
○ No 199

Skin examination by yourself or a friend

○ Yes Year [ ]
○ No 199

Test for blood in faeces (bowel motions)

○ Yes Year [ ]
○ No 199

Rectal examination (doctor uses a finger to check the back passage)

○ Yes Year [ ]
○ No 199

Have you had any of the following examinations or tests *in the last 5 years?*

Sigmoidoscopy (doctor uses a tube to examine lower bowel)

○ Yes Year
○ No 199 ☐ ⓪①②③④⑤⑥⑦⑧⑨

Colonoscopy (doctor uses a long tube to examine the whole bowel)

○ Yes Year
○ No 199 ☐ ⓪①②③④⑤⑥⑦⑧⑨

Barium Meal (X-ray of stomach after drinking barium)

○ Yes Year
○ No 199 ☐ ⓪①②③④⑤⑥⑦⑧⑨

Barium Enema (X-ray of bowel after having a barium enema)

○ Yes Year
○ No 199 ☐ ⓪①②③④⑤⑥⑦⑧⑨

Chest X-ray

○ Yes Year
○ No 199 ☐ ⓪①②③④⑤⑥⑦⑧⑨

CAT Scan / MRI Scan

○ Yes Year
○ No 199 ☐ ⓪①②③④⑤⑥⑦⑧⑨

MEN ONLY

Blood test for PSA (prostate specific antigen)

○ Yes Year
○ No 199 ☐ ⓪①②③④⑤⑥⑦⑧⑨

WOMEN ONLY

Vaginal examination by a doctor

○ Yes Year
○ No 199 ☐ ⓪①②③④⑤⑥⑦⑧⑨

Pap test (Pap smear)

○ Yes Year
○ No 199 ☐ ⓪①②③④⑤⑥⑦⑧⑨

Mammogram

○ Yes Year
○ No 199 ☐ ⓪①②③④⑤⑥⑦⑧⑨

Breast examination by a doctor

○ Yes Year
○ No 199 ☐ ⓪①②③④⑤⑥⑦⑧⑨

Breast self examination

○ Yes Year
○ No 199 ☐ ⓪①②③④⑤⑥⑦⑧⑨

SECTION 6:
PAIN KILLERS

On average, how many tablets or capsules do you take of the following types of pain killer?

To help you, please look at the list on the back of this booklet to see what names are included in each of the categories.

	none or less than 1 per week	1 or 2 per week	3 to 5 per week	about 1 per day	more than 1 per day
PARACETAMOL	○	○	○	○	○
ASPIRIN 300 mg	○	○	○	○	○
ASPIRIN 100 mg	○	○	○	○	○
NSAIDs (non-steroidal anti-inflammatory drugs)	○	○	○	○	○

If you currently use any of the above regularly (at least once per week), for about how many years have you used them?

	I don't use regularly	less than 1 year	1 to 2 years	3 to 5 years	more than 5 years
PARACETAMOL	○	○	○	○	○
ASPIRIN	○	○	○	○	○
NSAIDs	○	○	○	○	○

SECTION 7:
SMOKING

1 **Over the last 5 years have you smoked cigarettes at all?**

○ No ► Go to SECTION 8 ○ Yes

2 **Do you currently smoke cigarettes?**

○ No ○ Yes ► Go to Question 5

3 **How many years ago did you quit?**

○ Less than 1 year
○ 1 ○ 2 ○ 3 ○ 4 ○ 5 years

4 **How many cigarettes did you smoke each day in the year before you quit?**

○ 1 - 4 ○ 15 - 24 ○ 40 or more
○ 5 - 14 ○ 25 - 39

► Go to SECTION 8

5 **On average how many cigarettes do you smoke each day?**

○ 1 - 4 ○ 15 - 24 ○ 40 or more
○ 5 - 14 ○ 25 - 39

SECTION 8:

GENERAL ITEMS

1 **How much do you weigh now?**

○ Don't know

Kilos

	0	0
1	1	1
2	2	2
	3	3
	4	4
	5	5
	6	6
	7	7
	8	8
	9	9

OR

Stones / Pounds

Stones		Pounds	
	0	0	10
1	1	1	11
2	2	2	12
3	3	3	13
4	4	4	
	5	5	
	6	6	
	7	7	
	8	8	
	9	9	

2 **What is your waist size?** While standing, please measure to the nearest centimetre using a tape measure placed at the level of your navel - do not pull in your stomach.

○ Don't know

cms

	0	0
1	1	1
2	2	2
	3	3
	4	4
	5	5
	6	6
	7	7
	8	8
	9	9

OR

inches

0	0
1	1
2	2
3	3
4	4
5	5
6	6
7	7
8	8
9	9

3 **With which hand do you usually?**

write ○ left ○ either ○ right
throw a ball ○ left ○ either ○ right

4 **Employment status**

○ employed, less than 20 hours per week
○ employed, 20 or more hours per week
○ home duties
○ unemployed
○ retired
○ student

SECTION 9:

FOOD AND DRINK

1 **What *one* type of bread do you eat most often?**

○ fibre increased white bread (eg. Hifibre)
○ white bread
○ wholemeal bread
○ multi-grain bread
○ other, please specify ______________
○ I don't eat bread ▶ Go to question 5

2 **On a typical day, how many slices of bread do you usually eat?** (count one bread roll as 2 slices)

○ 1 slice per day
○ 2 slices per day
○ 3 slices per day
○ 4 slices per day
○ 5 - 7 slices per day
○ 8 or more slices per day

3 **Which *one* of the following do you most often put on bread?**

○ polyunsaturated margarine (made from sunflower, safflower or corn oil)
○ monounsaturated margarine (made from canola or olive oil)
○ butter and margarine blends
○ butter
○ olive oil ▶ Go to question 5
○ I don't usually put any oil or fat spread on bread ▶ Go to question 5

4 **How do you usually spread butter or margarine on a slice of bread?**

○ thickly
○ medium (the amount in a single wrapped portion in restaurants)
○ thinly

5 **How many eggs do you usually eat each week?**

○ less than 1 egg per week
○ 1 - 2 eggs per week
○ 3 - 5 eggs per week
○ 6 or more eggs per week
○ I don't eat eggs

6 **How many serves of fruit do you usually eat each day?** (a "serve" = 1 medium piece or 2 small pieces of fruit or 1 cup of diced pieces)

○ 1 serve or less
○ 2 - 3 serves
○ 4 - 5 serves
○ 6 serves or more
○ I don't eat fruit

7 **How many serves of vegetables do you usually eat each day?** (a "serve" = 1/2 cup cooked vegetables or 1 cup of salad vegetables)

○ 1 serve or less
○ 2 - 3 serves
○ 4 - 5 serves
○ 6 serves or more
○ I don't eat vegetables

8 **How much salt is added during cooking to the vegetables you eat at home?**

- ○ none
- ○ just a little
- ○ a moderate amount
- ○ quite a lot

9 **When available, how often do you choose foods labelled low salt, salt reduced or no added salt?**

- ○ never
- ○ occasionally
- ○ about half the time
- ○ mostly
- ○ always

10 **When you eat grilled or roast meat, how is it usually cooked?**

- ○ rare
- ○ medium rare
- ○ medium
- ○ well done
- ○ very well done
- ○ I don't eat meat ► Go to question 12

11 **On average during the last year, how often did you eat meat that had been cooked in the following ways?**

	never	less than once per month	1 to 3 times per month	once per week	twice per week	3 or more times per week
Casseroled or stewed	○	○	○	○	○	○
Grilled	○	○	○	○	○	○
Roasted	○	○	○	○	○	○
Fried or stir fried	○	○	○	○	○	○
Barbequed	○	○	○	○	○	○

12 **What one type of milk do you drink or use most often?**

- ○ Full cream milk
- ○ Reduced fat milk (eg REV, PhysiCAL)
- ○ Skim milk
- ○ Soya milk
- ○ Other ______________
- ○ I don't drink milk ►Go to question 14

13 **How much milk do you usually drink each day (including milk with tea, coffee, cereal etc)?**

- ○ Less than 250 ml (1 large cup or mug)
- ○ 250 - 500 ml (1-2 cups)
- ○ 500 - 750 ml (2-3 cups)
- ○ 750 ml (3 cups) or more

14 **For each food, indicate HOW MUCH ON AVERAGE you usually eat at a meal by marking *one* of the bubbles (○) below the pictures. If you eat two or more helpings, count them altogether.**

When you eat steak, do you usually eat: ○ I never eat steak

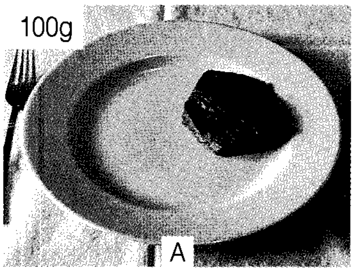

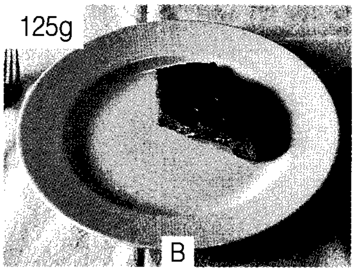

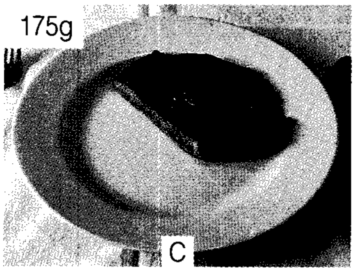

○ Less than A ○ A ○ Between A & B ○ B ○ Between B & C ○ C ○ More than C

When you eat casserole, do you usually eat: ○ I never eat casserole

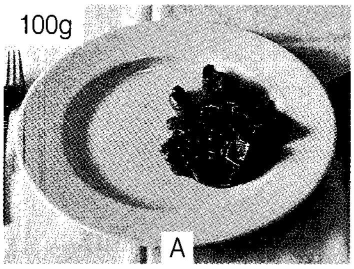

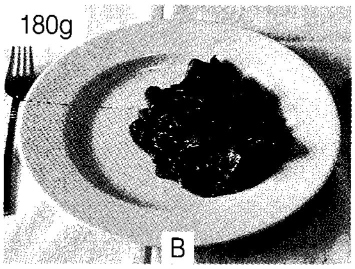

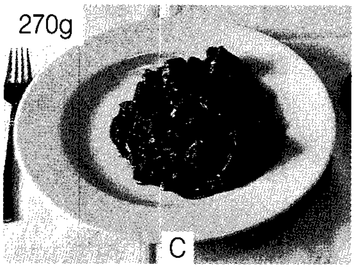

○ Less than A ○ A ○ Between A & B ○ B ○ Between B & C ○ C ○ More than C

When you eat rice, do you usually eat: ○ I never eat rice

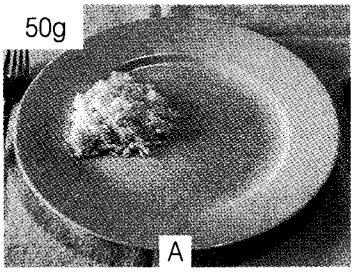

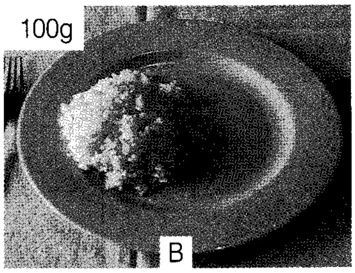

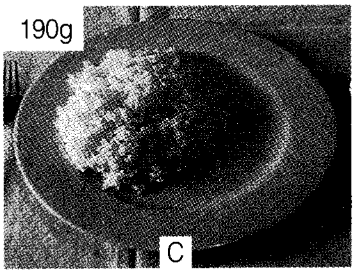

○ Less than A | ○ A | ○ Between A & B | ○ B | ○ Between B & C | ○ C | ○ More than C

When you eat potato, do you usually eat: ○ I never eat potato

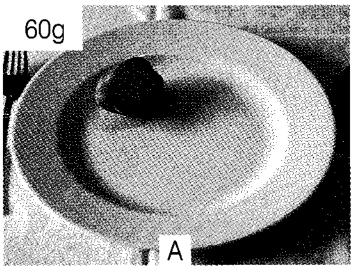

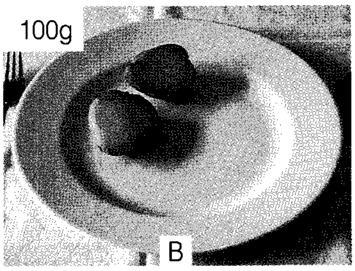

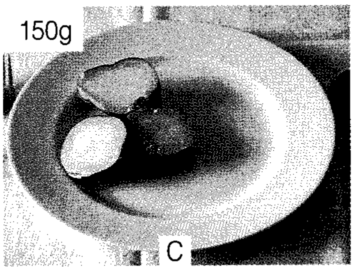

○ Less than A | ○ A | ○ Between A & B | ○ B | ○ Between B & C | ○ C | ○ More than C

When you eat vegetables, do you usually eat: ○ I never eat vegetables

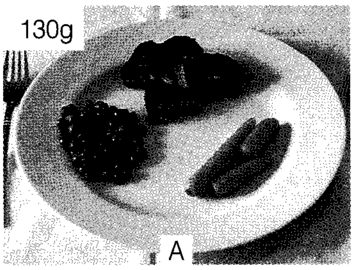

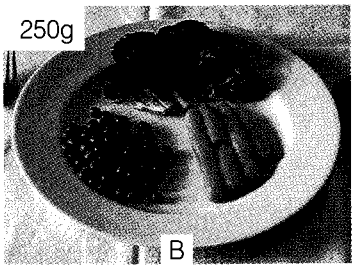

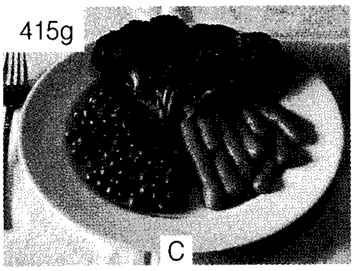

○ Less than A | ○ A | ○ Between A & B | ○ B | ○ Between B & C | ○ C | ○ More than C

When you eat salad, do you usually eat: ○ I never eat salad

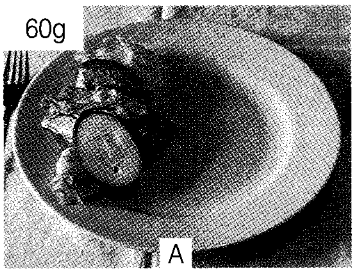

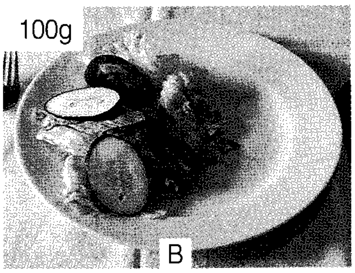

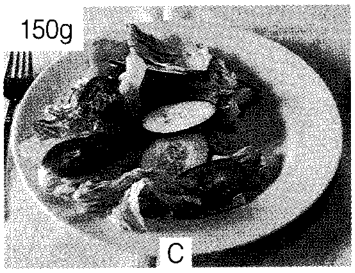

○ Less than A | ○ A | ○ Between A & B | ○ B | ○ Between B & C | ○ C | ○ More than C

15 **On average, how many cups or glasses of the following did you drink over the last year.** As the amount of some drinks will vary according to the weather, give the amount that you would drink during moderate conditions, neither too hot nor too cold. **(mark only one answer per line)**

Average number of **cups or glasses** you drank over the last year	**never**	**per month**		**per week**			**per day**			
		less than 1	1 - 3	1	2 - 4	5 - 6	1	2 - 3	4 - 5	6 or more
water	○	○	○	○	○	○	○	○	○	○
tea (including herbal tea)	○	○	○	○	○	○	○	○	○	○
coffee	○	○	○	○	○	○	○	○	○	○
fruit juice or drink	○	○	○	○	○	○	○	○	○	○
low alcohol beer	○	○	○	○	○	○	○	○	○	○
full strength beer	○	○	○	○	○	○	○	○	○	○
red wine	○	○	○	○	○	○	○	○	○	○
white wine (inc. sparkling)	○	○	○	○	○	○	○	○	○	○
spirits, liqueurs, sherry, port	○	○	○	○	○	○	○	○	○	○

16 **Over the last year, how often did you eat the following foods, ingredients or supplements? (mark only one answer per line)**

Average number of **times** you consumed these foods over the last year	**never**	**per month**		**per week**				**per day**	
		less than 1	1 - 3	1	2	3 - 4	5 - 6	1	2 or more
All Bran	○	○	○	○	○	○	○	○	○
Sultana Bran, Fibreplus, Branflakes	○	○	○	○	○	○	○	○	○
Weet Bix, Vita Brits, Weeties	○	○	○	○	○	○	○	○	○
Cornflakes, Nutrigrain, Special K	○	○	○	○	○	○	○	○	○
oatmeal porridge	○	○	○	○	○	○	○	○	○
muesli	○	○	○	○	○	○	○	○	○
citrus fruit, eg. oranges, in summer	○	○	○	○	○	○	○	○	○
citrus fruit, eg. oranges, in winter	○	○	○	○	○	○	○	○	○
fresh tomatoes in summer	○	○	○	○	○	○	○	○	○
fresh tomatoes in winter	○	○	○	○	○	○	○	○	○
green salad in summer	○	○	○	○	○	○	○	○	○
green salad in winter	○	○	○	○	○	○	○	○	○
salad in a sandwich or roll	○	○	○	○	○	○	○	○	○
dried beans, peas or lentils	○	○	○	○	○	○	○	○	○
fish, tinned or fresh	○	○	○	○	○	○	○	○	○
chicken	○	○	○	○	○	○	○	○	○
veal	○	○	○	○	○	○	○	○	○
beef	○	○	○	○	○	○	○	○	○
lamb	○	○	○	○	○	○	○	○	○
parmesan cheese	○	○	○	○	○	○	○	○	○
garlic	○	○	○	○	○	○	○	○	○
chilli	○	○	○	○	○	○	○	○	○
polyunsaturated salad/cooking oil	○	○	○	○	○	○	○	○	○
canola oil	○	○	○	○	○	○	○	○	○
olive oil	○	○	○	○	○	○	○	○	○
multivitamins - tablets, capsules	○	○	○	○	○	○	○	○	○
vitamin C - tablets, capsules	○	○	○	○	○	○	○	○	○
vitamin E - tablets, capsules	○	○	○	○	○	○	○	○	○

17 **If you take vitamins, minerals or other dietary supplements regularly, please write the brands and doses below.**

______________________ __________ ______________________________

______________________ __________ ______________________________

______________________ __________ ______________________________

SECTION 10:
LIFESTYLE

Questions 1 to 3 relate to **the last 12 months.**

1 How many times a week did you walk for at least 20 minutes, or at least one kilometre for recreation or exercise?

- ○ none
- ○ less than once per week
- ○ once or twice per week
- ○ 3 - 5 times per week
- ○ 6 or more times per week

2 How many times a week did you engage in more vigorous exercise than walking for at least 20 minutes?

- ○ none
- ○ less than once per week
- ○ once or twice per week
- ○ 3 - 5 times per week
- ○ 6 or more times per week

3 During the last 12 months, to what extent:

	not at all	a little bit	moderately	quite a bit	extremely
Did health problems limit you in your everyday physical activity (such as walking and climbing stairs)?	○	○	○	○	○
Did pain interfere with your normal work (including both work outside the house and housework)?	○	○	○	○	○
Has your physical health or emotional problems interfered with your normal social activities with family, friends, or groups?	○	○	○	○	○
Have you been bothered by emotional problems (such as feeling anxious, depressed or irritable)?	○	○	○	○	○
Was it difficult doing your daily work, both inside and outside the house, because of your physical health or emotional problems?	○	○	○	○	○

4 In general, how would you describe your health?

- ○ poor
- ○ fair
- ○ good
- ○ very good
- ○ excellent

5 To what extent do you agree or disagree with the following statement: "I'm always optimistic about my future".

- ○ strongly agree
- ○ agree
- ○ neither agree nor disagree
- ○ disagree
- ○ strongly disagree

6 Including yourself, how many people live in your household?

- ○ I live alone
- ○ 2 people
- ○ 3 or 4 people
- ○ 5 or more people

7 On average, how often do you visit friends and relatives?

- ○ once per month or less
- ○ 2 - 3 times per month
- ○ about once per week
- ○ 2 - 3 times per week
- ○ 4 - 6 times per week
- ○ every day

8 On average, how often do friends and relatives visit you at your home?

- ○ once per month or less
- ○ 2 - 3 times per month
- ○ about once per week
- ○ 2 - 3 times per week
- ○ 4 - 6 times per week
- ○ every day

9 Do you have someone who makes you feel needed?

- ○ Yes, very much
- ○ Yes, somewhat
- ○ No, not really

SECTION 11: QUESTIONS FOR MEN ONLY

1 **For how many children have you been the biological father?**

Boys ○ none ○ 1 ○ 2 ○ 3 ○ 4 ○ 5 or more

Girls ○ none ○ 1 ○ 2 ○ 3 ○ 4 ○ 5 or more

2 **Have you had a vasectomy?**

○ No ○ Yes

If Yes, in what year?

Year 19 ☐☐

⑤ ⑥ ⑦ ⑧ ⑨

⓪ ① ② ③ ④ ⑤ ⑥ ⑦ ⑧ ⑨

3 **Over the past month, how often have you:**

	not at all	less than 1 time in 5	less than half the time	about half the time	more than half the time	almost always
Found it difficult to postpone urination?	○	○	○	○	○	○
Had to push or strain to begin urination?	○	○	○	○	○	○
Had a weak urinary stream?	○	○	○	○	○	○
Found you stopped and started again several times when you urinated?	○	○	○	○	○	○
Had a sensation of not emptying your bladder completely after you finished urinating?	○	○	○	○	○	○
Had to urinate again less than two hours after you finished urinating?	○	○	○	○	○	○

4 **Over the past month, how many times did you most typically get up from bed to urinate during the night?**

○ never
○ some nights
○ once per night
○ 2 times per night
○ 3 times per night
○ 4 times per night
○ 5 or more times per night

5 **Has a doctor ever examined your prostate?**

○ No ○ Don't know
○ Yes, I was first examined in

Year 19 ☐☐

⑤ ⑥ ⑦ ⑧ ⑨

⓪ ① ② ③ ④ ⑤ ⑥ ⑦ ⑧ ⑨

6 **Has a doctor ever told you that you have an enlarged prostate (or BPH)?**

○ No ○ Yes ○ Don't know

If Yes, in what year?

Year 19 ☐☐

⑤ ⑥ ⑦ ⑧ ⑨

⓪ ① ② ③ ④ ⑤ ⑥ ⑦ ⑧ ⑨

7 **Have you ever had a PSA test?**

○ No ▶ Go to question 8
○ Don't know
○ Yes, I had my first PSA test in

Year 19 ☐☐

⑧ ⑨

⓪ ① ② ③ ④ ⑤ ⑥ ⑦ ⑧ ⑨

Why did you have your first PSA test?

○ because of urinary symptoms
○ as a health check up

How many times altogether have you had a PSA test?

○ 1 ○ 2 ○ 3 ○ 4 ○ 5 or more

8 **Have you ever had treatment for prostate problems?**

○ No ○ Yes

If **Yes,** please complete the details on the yellow sheet provided.

9 **Has a male relative of yours ever had prostate cancer?**

(include your father, grandfathers, your mother's brothers, your father's brothers, your brothers and sons)

○ No ○ Yes ○ Don't know

If **Yes,** give their relationship to you and their age at diagnosis (if known).

Relative ________________ Age ________

Relative ________________ Age ________

SECTION 12: QUESTIONS FOR WOMEN ONLY

1 **Have you ever given birth to twins, triplets or quads?**

○ No ○ Yes

2 **Over the last 5 years have you had any pregnancies (including any miscarriages or terminations)?**

○ No ○ Yes

If **Yes,** please give details for each pregnancy on the yellow sheet of paper.

3 **Over the last 5 years for how long, if at all, have you used the contraceptive pill?**

○ Not at all ► Go to question 4
○ less than a year
○ 1 ○ 2 ○ 3 ○ 4 ○ 5 years

Do you currently take the pill?

○ No ○ Yes

What was the name of the pill you last used (or still use)?

____

4 **Over the last 5 years for how long, if at all, have you used hormone replacement therapy (HRT)?**

HRT includes female hormone medications such as oestrogen replacement pills, patches, implants or injections.

○ Not at all ► Go to question 5
○ less than a year
○ 1 ○ 2 ○ 3 ○ 4 ○ 5 years

If used at all, what types of HRT have you used? (mark all that apply)

○ Pills (one type per month)
○ Pills (two types per month)
○ Patches
○ Pills and patches during each month
○ Injections
○ Implants (under the skin)

Do you currently take **HRT?**

○ No ○ Yes

Please write down the name(s) of the **HRT** you last used or still use

____

____

5 **Have you had a menstrual period in the last 12 months?**

○ No ► Go to question 6
○ Yes

If **Yes,** are your periods as regular now as they were up to the age of 40?

○ No ○ Yes ○ Don't know

6 **In what year was your last period?**

My last period was in

Year 19 [ ][ ]

			③	④	⑤	⑥	⑦	⑧	⑨
⓪	①	②	③	④	⑤	⑥	⑦	⑧	⑨

7 **Have you had a hysterectomy?**

○ No ○ Yes

If Yes, in what year?

Year 19 [ ][ ]

			③	④	⑤	⑥	⑦	⑧	⑨
⓪	①	②	③	④	⑤	⑥	⑦	⑧	⑨

8 **Have you had any of your ovaries removed?**

○ No ○ Yes

If Yes, in what year?

Year 19 [ ][ ]

			③	④	⑤	⑥	⑦	⑧	⑨
⓪	①	②	③	④	⑤	⑥	⑦	⑧	⑨

How many ovaries were removed?

○ one ○ both ○ Don't know

9 **Has a female relative of yours ever had breast cancer?**

(include your mother, grandmothers, your mother's sisters, your father's sisters, your sisters and daughters)

○ No ○ Yes ○ Don't know

If **Yes,** give their relationship to you and their age at diagnosis (if known).

Relative ________________ Age _______

Relative ________________ Age _______

PARACETAMOL PREPARATIONS

Dymadon
Panadol
Panamax
Paraspen
Setamol
Tempra
Tylenol

Paracetamol mixtures (count as Paracetamol)
Codral
Dymadon Co
Fiorinal
Mersyndol
Panadeine
Panalgesic
Panamax
Panquil
Panadol Elixir

ASPIRIN PREPARATIONS

Aspirin 300 mg
Aspirin
Aspro
Bex
Bufferin
Disprin
Ecotrin
Solprin
Spren
Vincents Powder
Winsprin

Aspirin Mixtures (count as Aspirin 300 mg)
Codiphen
Codis
Codox
Decrin
Disprin Forte
Solcode
Veganin

Aspirin 100 mg
Cardiprin 100
Astrix 100
Cartia

NON-STEROIDAL ANTI-INFLAMMATORY DRUGS (NSAIDS)

Aclin
Act -3
Anaprox
Arthrexin
Brufen
Butazolidin
Clinoril
Dolobid
Feldene
Fenac
Indocid
Inza
Mefic
Naprogesic
Naprosyn
Nurofen
Orudis
Oruvail SR
Ponstan
Proxen SR
Rafen
Surgam
Synflex
Tilcotil
Voltaren
Voltaren Rapid

Anti-Cancer Council
of Victoria

© ACCV 1995
1 Rathdowne Street, Carlton Victoria 3053
