# Supplementary material for: Prospective Evaluation over 15 Years of Six Breast Cancer Risk Models
Source: Cancers (Basel). 2021 Oct 16;13(20):5194. doi: 10.3390/cancers13205194 (PMC8534072; doi:10.3390/cancers13205194)
Supplement: Supplementary file 1 [file cancers-13-05194-s001.zip › MCCS_Questionnaire_FUP2.pdf]

Health & Lifestyle Questionnaire – English V15

H2ID |___| |___| |___| |___| |___|

Last name ____________________

D.O.B. ______________________

HEALTH 2020

A study of lifestyle and health

The Cancer Council Victoria

Leading the fight...

Information for interviewers…

Questions and statements in regular bold font are read

Statements and interviewer prompts in regular font are read if necessary

Statements, instructions and responses in italics are not read

Date |__|__| / |__|__|/ |__|__|__|__|

Time interview started: |__|__|: |__|__| AM PM (Please circle)

Interviewer name: ______________________________

Interviewer code: |__|__|

STRT

Interviewer question: *(DO NOT READ THIS QUESTION, please select an option)*

How is the respondent being interviewed?

1 Telephone interview before clinical assessment

2 Telephone interview after clinical assessment

INTROA

Good morning/afternoon/ evening, my name is *{your name}* **from Health 2020. May I please speak with** *{Participants title, participants last name}*?

I am calling for the appointment we made with you on *{Month, day}* **over the telephone. I'd like to start by confirming that I am speaking with the correct person please? Can you please tell me your date of birth? What is your address?** *(INTERVIEWER TO VERIFY)*

Thank you.

INTROA1

INTERVIEWER INSTRUCTION:
PRESS RECORD NOW

{Participants title, partipants last name}, **I'd just like to let you know that our interviews may be monitored for quality control purposes.**

Your reference number is_____________, my name is__________________________

and I will be interviewing you today, on the________________, at_______________.

INTERVIEWER INSTRUCTION:
If participant objects to being recorded STOP recording now.

If telephone interview before clinical assessemnt	*Go to INTRO1*
If telephone interview after clinical assessment	*Go to BGIN*

INTRO1

Do you have the pink showcard with you? The showcard has a list of possible answers to most of the questions I will ask you today to make the interview easier.

INTERVIEWER CLARIFICATION:
This is a piece of pink A4 paper which was given to you by the person who collected your blood.

1 Yes

2 No

BGIN

Just before we begin I'd like to let you know that some of the questions that I ask you today might seem repetitive. For our research, it is important that I ask everyone all the questions in exactly the same way. There are no right or wrong answers to any of the questions. If you do not understand any of the questions please tell me.

Some of the questions refer to a scale for the answers.

[If YES to showcard]
I will ask you to refer to the show cards that we gave you. For those questions please choose the closest answer using a number or the statement. Okay?

OR

[If NO to showcard]
I will read out the scale to you. For those questions please choose the closest answer using a number or the statement. Okay?

Are you ready to start?

DIET

Let's start with a question about your diet.

1. How much have you changed your diet in the last 5 years?

READ CHOICES

1 Not at all

2 A little bit

3 Moderately

4 A lot

8 Refused (DO NOT READ)

9 Don't Know/Not sure (DO NOT READ)

SECTION 1 SOCIAL AND SUPPORT

LIVWTH

2. Who do you currently live with?

INTERVIEWER INSTRUCTION
Enter all that apply

DO NOT READ CHOICES

1 No one *Go to Question 3*

2 Your husband, wife or partner

3 Your parent(s) or spouse/partners parent(s)

4 Your child(ren) If yes, Please tell me the ages of the child(ren) you live with?

5 Your grandchild(ren) If yes, Please tell me the ages of the child(ren) you live with*?*

6 Shared accommodation (eg hostel, communal retirement village)

7 Someone else. If yes, Please tell me who else you currently live with?

8 Refused (DO NOT READ)

9 Don't know/Not sure (DO NOT READ)

CHILDAGE

Use the lines below to record the additional information provided with question 2

Age of child(ren)	*Age of grandchild(ren)*

Record response here if the respondent lives with someone else.

____

VISITFR

3. On average, how often do you visit friends and relatives?

1 *Once per month or less*
2 *2-3 times per month*
3 *About once per week*
4 *2-3 times per week*
5 *4-6 times per week*
6 *7 times per week*
8 *Refused* *(DO NOT READ)*
9 *Don't Know/Not Sure* *(DO NOT READ)*

FRVISIT

4. On average, how often do friends and relatives visit you at your home?

1 *Once per month or less*
2 *2-3 times per month*
3 *About once per week*
4 *2-3 times per week*
5 *4-6 times per week*
6 *7 times per week*
8 *Refused* *(DO NOT READ)*
9 *Don't Know/Not Sure* *(DO NOT READ)*

SOCIALA

5. How many hours a week, if any, do you spend involved in social activities outside your home or work? (for example, at social clubs, work clubs, church groups or sporting groups and other community groups or regular social meetings with friends)

1 *None*
2 *1 or 2*
3 *3 to 4*
4 *5 to 9*
5 *10 or more*
8 *Refused* *(DO NOT READ)*
9 *Don't Know/Not Sure* *(DO NOT READ)*

PETS

6. Do you have any pets at home?

1	*Yes*		
2	*No*		*GO TO NEXT SECTION*
8	*Refused*	*(DO NOT READ)*	*GO TO NEXT SECTION*
9	*Don't Know/Not Sure*	*(DO NOT READ)*	*GO TO NEXT SECTION*

DOG

7. Do you have a dog?

1	*Yes*	
2	*No*	
8	*Refused*	*(DO NOT READ)*
9	*Don't Know/Not Sure*	*(DO NOT READ)*

CAT

8. A cat?

1	*Yes*	
2	*No*	
8	*Refused*	*(DO NOT READ)*
9	*Don't Know/Not Sure*	*(DO NOT READ)*

SECTION 2 Quality of life - SF12v2, USA version

These questions are about your health.

HLTHQLTY

9. The next question asks for your views about your health. This information will help keep track of how you feel and how well you are able to do your usual activities.
In general, would you say your health is:

READ OUT CHOICES

1 Excellent

2 Very good

3 Good

4 Fair

5 Poor - *[Interviewer please take note of this response.]*

8 Refused (DO NOT READ)

9 Don't know/Not sure (DO NOT READ)

IMTMOD

10. The following questions are about activities you might do during a typical day. <u>Does your health now limit you</u> in these activities? If so, how much?

<u>Moderate activities</u> (such as moving a table, pushing a vacuum cleaner, bowling, or playing golf)?

READ OUT CHOICES

1 Yes, limited a lot

2 Yes, limited a little

3 No, not limited at all

8 Refused (DO NOT READ)

9 Don't know/Not sure (DO NOT READ)

IMTSTAR

11. Climbing <u>several</u> flights of stairs?

READ OUT CHOICES

1 Yes, limited a lot

2 Yes, limited a little

3 No, not limited at all

8 Refused (DO NOT READ)

9 Don't know/Not sure (DO NOT READ)

PHYLESS

[If YES to showcard] **Please refer to show card 3 to help you answer the next questions.**

12. During the past 4 weeks, how much of the time have you had any of the following problems with your work or other regular daily activities as a result of your physical health?

Accomplished less than you would like?

1 All of the time

2 Most of the time

3 Some of the time

4 A little of the time

5 None of the time

8 Refused (DO NOT READ)

9 Don't know/Not sure (DO NOT READ)

PHYLMT

13. Were limited in the kind of work or other activities?

DO NOT READ CHOICES UNLESS NECESSARY

1 All of the time

2 Most of the time

3 Some of the time

4 A little of the time

5 None of the time

8 Refused (DO NOT READ)

9 Don't know/Not sure (DO NOT READ)

MINDLESS

14. During the past 4 weeks, how much of the time have you had any of the following problems with your work or other regular daily activities as a result of any emotional problems, such as feeling depressed or anxious?

Accomplished less than you would like?

DO NOT READ CHOICES UNLESS NECESSARY

1 All of the time

2 Most of the time

3 Some of the time

4 A little of the time

5 None of the time

8 Refused (DO NOT READ)

9 Don't know/Not sure (DO NOT READ)

MINDCARE

15. Did work or other activities <u>less carefully than usual</u>?

DO NOT READ CHOICES UNLESS NECESSARY

1 All of the time

2 Most of the time

3 Some of the time

4 A little of the time

5 None of the time

8 Refused (DO NOT READ)

9 Don't know/Not sure (DO NOT READ)

PAINLMT

[If YES to showcard] **Please refer to show card 4 to help you answer the next question.**

16. During the <u>past 4 weeks</u>, how much did <u>pain</u> interfere with your normal work (including both work outside the home and housework)?

1 Not at all

2 A little bit

3 Moderately

4 Quite a bit

5 Extremely

8 Refused (DO NOT READ)

9 Don't know/Not sure (DO NOT READ)

FEELCALM

[If YES to showcard] **Please refer to show card 3 to help you answer the next questions.**

17. These questions are about how you feel and how things have been with you <u>during the past 4 weeks</u>. For each question, please give the one answer that comes closest to the way you have been feeling.

How much of the time during the <u>past 4 weeks</u>:

Have you felt calm and peaceful?

DO NOT READ CHOICES UNLESS NECESSARY

1 All of the time

2 Most of the time

3 Some of the time

4 A little of the time

5 None of the time

8 Refused *(DO NOT READ)*

9 Don't know/Not sure *(DO NOT READ)*

FEELERGY

18. Did you have a lot of energy?

DO NOT READ CHOICES UNLESS NECESSARY

1 All of the time

2 Most of the time

3 Some of the time

4 A little of the time

5 None of the time

8 Refused *(DO NOT READ)*

9 Don't know/Not sure *(DO NOT READ)*

FEELDOWN

19. Have you felt downhearted and depressed?

DO NOT READ CHOICES UNLESS NECESSARY

1 All of the time

2 Most of the time

3 Some of the time

4 A little of the time

5 None of the time

8 Refused *(DO NOT READ)*

9 Don't know/Not sure *(DO NOT READ)*

SOCPROB

20. During the past 4 weeks, how much of the time has your physical health or emotional problems interfered with your social activities (like visiting friends, relatives, etc.)?

DO NOT READ CHOICES UNLESS NECESSARY

1 All of the time

2 Most of the time

3 Some of the time

4 A little of the time

5 None of the time

8 Refused *(DO NOT READ)*

9 Don't know/Not sure *(DO NOT READ)*

SECTION 3 ACTIVITIES OF DAILY LIVING

INTERVIEWER INSTRUCTION:
If 'Poor' was answered at question 9, skip Activities of Daily Living, Smoking , Alcohol and Physical Activity section and go to question 66.

These questions are only asked of people 70 years of age or older.

[If YES to showcard] **Please refer to show card 5 to help you answer the next questions.**

HLTHBATH

Because of your health, do you have difficulty with …

21. Bathing yourself?

DO NOT READ CHOICES UNLESS NECESSARY

1 None
2 Some
3 A lot
4 Cannot do
8 Refused (DO NOT READ)
9 Don't know/Not sure (DO NOT READ)

HLTHDRES

22. Dressing?

DO NOT READ CHOICES UNLESS NECESSARY

1 None
2 Some
3 A lot
4 Cannot do
8 Refused (DO NOT READ)
9 Don't know/Not sure (DO NOT READ)

HLTHEAT

23. Eating?

DO NOT READ CHOICES UNLESS NECESSARY

1 None
2 Some
3 A lot
4 Cannot do
8 Refused (DO NOT READ)
9 Don't know/Not sure (DO NOT READ)

HLTHBED

24. Getting out of bed or a chair?

DO NOT READ CHOICES UNLESS NECESSARY

1 None

2 Some

3 A lot

4 Cannot do

8 Refused (DO NOT READ)

9 Don't know/Not sure (DO NOT READ)

HLTHTOIL

25. Going to and using the toilet at home?

DO NOT READ CHOICES UNLESS NECESSARY

1 None

2 Some

3 A lot

4 Cannot do

8 Refused (DO NOT READ)

9 Don't know/Not sure (DO NOT READ)

HLTHPH

26. Using the telephone?

DO NOT READ CHOICES UNLESS NECESSARY

1 None

2 Some

3 A lot

4 Cannot do

8 Refused (DO NOT READ)

9 Don't know/Not sure (DO NOT READ)

HLTHSHOP

27. Shopping?

DO NOT READ CHOICES UNLESS NECESSARY

1 None

2 Some

3 A lot

4 Cannot do

8 Refused (DO NOT READ)

9 Don't know/Not sure (DO NOT READ)

HLTHWLK

28. Walking about 200 to 300 metres (i.e. around the block)?

DO NOT READ CHOICES UNLESS NECESSARY

1 None

2 Some

3 A lot

4 Cannot do

8 Refused (DO NOT READ)

9 Don't know/Not sure (DO NOT READ)

HLTHOUT

29. Going out by car or bus, by yourself?

DO NOT READ CHOICES UNLESS NECESSARY

1 None

2 Some

3 A lot

4 Cannot do

8 Refused (DO NOT READ)

9 Don't know/Not sure (DO NOT READ)

HLTHFING

30. Picking up something with your fingers or using your fingers freely?

DO NOT READ CHOICES UNLESS NECESSARY

1 None

2 Some

3 A lot

4 Cannot do

8 Refused (DO NOT READ)

9 Don't know/Not sure (DO NOT READ)

HLTHBAG

31. Carrying a heavy shopping bag?

DO NOT READ CHOICES UNLESS NECESSARY

1 None

2 Some

3 A lot

4 Cannot do

8 Refused (DO NOT READ)

9 Don't know/Not sure (DO NOT READ)

HLTHSTAR

32. Going up stairs (two or three steps)?

DO NOT READ CHOICES UNLESS NECESSARY

1 None

2 Some

3 A lot

4 Cannot do

8 Refused (DO NOT READ)

9 Don't know/Not sure (DO NOT READ)

HLTHWRK

33. Doing heavy work around the house such as shovelling dirt or washing walls?

DO NOT READ CHOICES UNLESS NECESSARY

1 None

2 Some

3 A lot

4 Cannot do

8 Refused (DO NOT READ)

9 Don't know/Not sure (DO NOT READ)

SECTION 4 SMOKING

NOWSMK

34. The next questions are about smoking.

Do you <u>now</u> smoke at least seven cigarettes a week?

1	*Yes*		
2	*No*		*Go to question 36*
8	*Refused*	*(DO NOT READ)*	*Go to question 36*
9	*Don't Know/Not Sure*	*(DO NOT READ)*	*Go to question 36*

MANDAY

35. On average, how many cigarettes do you smoke each day?

NUMDAY

|__|__| *cigarettes per day* *(Don't know = 99)*

OR

If respondent is unable to answer because pattern varies from day to day ask,

On average, how many cigarettes do you smoke each week?

NUMWK

|__|__|__| *cigarettes per week* *(Don't know = 999)*

Go to NEXT SECTION – question 38

LFETME

36. In the past, have you <u>ever</u> smoked at least 7 cigarettes a week?

1	*Yes*		
2	*No*		*Go to NEXT SECTION – question 38*
8	*Refused*	*(DO NOT READ)*	*Go to NEXT SECTION – question 38*
9	*Don't Know/Not Sure*	*(DO NOT READ)*	*Go to NEXT SECTION – question 38*

FINSTOP

37. When did you <u>last</u> stop smoking at least 7 cigarettes a week?

Use the options below to fill in the answer given by the respondent

FINSTOP

|__|__|*(day)* / |__|__|*(month)* / |__|__|__|__|*(year)*

OR

WKSAGO/MTHSAGO/YRSAGO

|__|__|*Weeks* |__|__| *Months* |__|__| *years ago*

OR

AGESTP

|__|__| *years old*

(Don't know = 99)

SECTION 5 ALCOHOL

DRINKal

The next questions are about alcohol.

38. In the last 12 months, how <u>often</u> did you drink <u>any</u> kind of alcoholic drink? This includes beer, wine, spirits, port, sherry or any drink containing alcohol.

If necessary, prompt with,
In the last 12 months, on how many days per month or week did you drink any kind of alcoholic drink?

If answer is given as number of times per month or number of times per week (eg twice a week), prompt for number of days instead.

How many <u>days</u> per week would that be?

DO NOT READ CHOICES

01 1 day per month or less often *Go to NEXT SECTION – question 48*

02 2 to 3 days per month

03 1 day per week

04 2 days per week

05 3-4 days per week

06 5-6 days per week

07 7 days per week

08 Never *Go to NEXT SECTION – question 48*

88 Refused (DO NOT READ)

99 Don't Know/Not Sure (DO NOT READ)

OFTBEER

39. In the last 12 months, how <u>often</u> did you drink beer?

If necessary, prompt with,
In the last 12 months, on how many days per month or week did you drink beer?

DO NOT READ CHOICES

01 1 day per month or less often *Go to question 42*

02 2 to 3 days per month

03 1 day per week

04 2 days per week

05 3-4 days per week

06 5-6 days per week

07 7 days per week

08 Never *Go to question 42*

88 Refused *(DO NOT READ)*

99 Don't Know/Not Sure *(DO NOT READ)*

UNTBEER

40. On the days when you drank beer during the last 12 months, how much did you <u>usually</u> drink in a single day?

Record exact number of drinks and unit of measure – e.g. 3 glasses, 0.5 (i.e. ½) bottle. Do not accept a range for the amount. If unsure of unit type/size, record in Other.

Amount |__|__|•|__|__|

Unit

1 Glasses ***(AMTBEERG)***

2 Pots ***(AMTBEERP)***

3 Stubbies ***(AMTBEERS)***

4 Cans ***(AMTBEERC)***

5 Bottles (prompt for size of the bottle eg is that a 750 mL bottle or a stubbie?) ***(AMTBEERB)***

6 Other ______________________________ *[Probe for container size in millilitres]*

8 Refused *(DO NOT READ)*

9 Don't Know/Not Sure *(DO NOT READ)*

LIGHTB

41. In the last 12 months, how much of the beer you drank was light beer? Light beer is sometimes called low alcohol beer.

INTERVIEWER CLARIFICATION: mid-strength beer is not light beer

READ OUT CHOICES

1 All or almost all

2 More than half

3 About half

4 Less than half

5 None or almost none

8 Refused *(DO NOT READ)*

9 Don't Know/Not Sure *(DO NOT READ)*

OFTWINE

42. In the last 12 months, how <u>often</u> did you drink wine?

[INTERVIEWER CLARIFICATION: By wine, I mean red wine, white wine, sparkling wine, champagne or rosé, but not fortified wines]

If necessary, prompt with,

In the last 12 months, on how many days per month or week did you drink wine?

DO NOT READ CHOICES

01 1 day per month or less often,		*Go to Question 45*
02 2 to 3 days per month		
03 1 day per week		
04 2 days per week		
05 3-4 days per week		
06 5-6 days per week		
07 7 days per week		
08 Never		*Go to question 45*
88 Refused	*(DO NOT READ)*	
99 Don't Know/Not Sure	*(DO NOT READ)*	

UNTWINE

43. On the days when you drank wine during the last 12 months, how much did you <u>usually</u> drink in a single day?

Record exact number of drinks and unit of measure – e.g. 3 glasses, 0.5 (i.e. ½) bottle. Do not accept a range for the amount. If unsure of unit type/size, record in Other.

Amount |__|__|•|__|__|

Unit

1 Glasses		***(AMTWINEG)***
2 Bottles		***(AMTWINEB)***
3 Litres		***(AMTWINEL)***
4 Other ______________________________ *[Probe for container size in millilitres]*		
8 Refused	*(DO NOT READ)*	
9 Don't Know/Not Sure	*(DO NOT READ)*	

REDWINE

44. In the last 12 months, how much of the wine you drank was red wine?

[INTERVIEWER CLARIFICATION: rose is not counted as red wine]

1 All or almost all

2 More than half

3 About half

4 Less than half

5 None or almost none

8 Refused *(DO NOT READ)*

9 *Don't Know/Not Sure* *(DO NOT READ)*

OFTSPIR

45. In the last 12 months, how <u>often</u> did you drink spirits? Spirits include whisky, gin, vodka, brandy, grappa, rum, on their own or as mixed drinks.

If necessary, prompt with,
In the last 12 months, on how many days per month or week did you drink spirits?

DO NOT READ CHOICES

01 1 day per month or less often *Go to NEXT SECTION – question 48*

02 2 to 3 days per month

03 1 day per week

04 2 days per week

05 3-4 days per week

06 5-6 days per week

07 7 days per week

08 Never *Go to NEXT SECTION – question 48*

88 Refused *(DO NOT READ)*

99 Don't Know/Not Sure *(DO NOT READ)*

UNTSP

46. On the days when you drank spirits during the last 12 months, how much did you <u>usually</u> drink in a single day?

INTERVIEWER CLARIFICATION
One Glass is equal to one standard mixed drink.

INTERVIEWER INSTRUCTION:
Record exact number of drinks and unit of measure – e.g. 3 glasses, ½ bottle. If unsure of unit type/size, record in Other and probe for millilitres.

Amount |__|__|•|__|__|

Unit

1	*Glasses*	*Go to NEXT SECTION*	*(AMTSPG)*
2	*Nips/Shots*	*Go to NEXT SECTION*	*(AMTSPN)*
3	*Cans*	*Go to NEXT SECTION*	*(AMTSPC)*
4	*Bottles*		*(AMTSPB)*
5	*Other* ______________________________	*– [Probe for container size in millilitres]*	
8	*Refused*	*(DO NOT READ)*	
9	*Don't Know/Not Sure*	*(DO NOT READ)*	

BOTSIZE

47. What size was the bottle?

|__|__|__|__| *millilitres* *(Don't know = 999)*

SECTION 6 PHYSICAL ACTIVITY

Modified from Long Last 7 Days Telephone IPAQ November 2002 revision

PACT

The next questions are about physical activity.

I am going to ask you about the time you spent being physically active in the last three months. Please answer each question even if you do not consider yourself to be an active person.

I will ask you about the activities you did at work, in your home, in your garden or yard, to get from place to place, to do errands and in your spare time for recreation, exercise or sport.

PART 1: JOB-RELATED PHYSICAL ACTIVITY

WRKVOL

48. The first questions are about your work. This includes paid jobs, including jobs where you work from home, and volunteer work that you did away from your home.

[INTERVIEWER CLARIFICATION: This does not include unpaid house or work in the garden or yard, nor caring for dependents; this will be asked in a later section.]

Do you currently have a job or do any volunteer work away from your home?

1	*Yes*		
2	*No*		*Go to question 53*
8	*Refused*	*(DO NOT READ)*	*Go to question 53*
9	*Don't Know/Not Sure*	*(DO NOT READ)*	*Go to question 53*

WRKUNT

49. On how many days per week do you usually work for pay or do volunteer work away from your home?

WRKDAYW

|__| days per week

8	*Refused*	*Go to PART 2 of Physical Activity Section - Go to question 53*
9	*Don't Know/Not Sure*	*Go to PART 2 of Physical Activity Section - Go to question 53*

If person cannot answer this because the days vary too much from week to week, ask:

WRKDAYM

On how many days per month do you usually work for pay or do volunteer work away from your home?

|__| *days per month* *Go to question 51*

8 Refused *Go to PART 2 of Physical Activity Section - Go to question 53*

9 Don't Know/Not Sure *Go to PART 2 of Physical Activity Section - Go to question 53*

WRKHRW

50. And how many hours per week?

|__|__| *hours per week* *Go to question 52* ***OR***

If less than 4 hours per week Go to PART 2 of Physical Activity Section – Go to question 53

88 Refused *Go to PART 2 of Physical Activity Section - Go to question 53*

99 Don't know/Not sure *Go to PART 2 of Physical Activity Section -Go to question 53*

WRKHRM

51. And how many hours per month?

|__|__|__| *hours per month* *If less than 16 hours per month go to PART 2 of Physical Activity Section question 53*

888 Refused *Go to PART 2 of Physical Activity Section - Go to question 53*

999 Don't know/Not sure *Go to PART 2 of Physical Activity Section - Go to question 53*

PHYWORK

[If YES to showcard] **Please refer to show card 6 to help you answer the next question.**

52. How would you describe the level of physical activity that you usually do at work?

READ OUT

1 Mainly sitting

2 Mainly sitting with occasional walking and moving about to do tasks

3 Mainly on feet with some light carrying or lifting

4 Hard physical effort such as scrubbing floors, digging, heavy carrying or lifting

8 Refused *(DO NOT READ)*

9 Don't know/Not sure *(DO NOT READ)*

PART 2: HOUSEWORK, HOUSE MAINTENANCE AND CARING FOR FAMILY

GRDNVIG

53. The next questions are about activities that you do in the garden or yard.

Think about activities which take moderate or vigorous physical effort. Moderate activities make you breathe somewhat harder than normal and vigorous activities make you breathe much harder than normal. Examples of moderate and vigorous activities include carrying light loads, sweeping, washing windows, raking, heavy lifting, chopping wood or digging. Include only those activities that you did for at least 10 minutes at a time.

In a typical week during the last 3 months, on how many days did you do <u>moderate or vigorous</u> physical activities <u>in the garden or yard</u>?

|__|	*Days per week*		*[If **0**, Go to Question 55]*
8	*Refused*	*(DO NOT READ)*	*[Go to Question 55]*
9	*Don't Know/Not Sure*	*(DO NOT READ)*	*[Go to Question 55]*

GRDNVIGU

54. On the days when you did moderate or vigorous activities in the garden or yard, how much time did you usually spend doing them in a single day?

GRDNVIGT

|__|__|:|__|__| *Hours: minutes per day* *(8800 = refused, 9900 = don't know)*

IF NECESSARY prompt with: An average time per day is being sought.

INTERVIEWER CLARIFICATION:
If the respondent can't answer because the pattern of time spent varies widely from day to day, ask:

What is the total amount of time you spent in a typical week over the last 3 months doing moderate or vigorous physical activities in the garden or yard?

|__|__|:|__|__| *Hours: minutes per week* *(8800 = refused, 9900 = don't know)*

HOMEMOD

55. The next questions are about chores or jobs that you do inside your home.

Think about activities which take at least moderate physical effort that you did inside your home. Examples of moderate activities may include carrying light loads, washing windows, scrubbing floors, vacuuming and sweeping. Include only those moderate or vigorous physical activities that you did for at least 10 minutes at a time.

In a typical week during the last 3 months, on how many days per week did you do at least moderate activities inside your home?

|__| *Days per week* *[If **0**, Go to Question 57]*

8 Refused (DO NOT READ) [Go to Question 57]

9 Don't Know/Not Sure (DO NOT READ) [Go to Question 57]

HOMEMODU

56. On the days when you did at least moderate activities inside your home, how much time did you usually spend doing them in a single day?

HOMEMODT

|__|__|:|__|__| *Hours: minutes per day (8800 = refused, 9900 = don't know)*

IF NECESSARY prompt with: An average time per day is being sought.

INTERVIEWER CLARIFICATION:
If the respondent can't answer because the pattern of time spent varies widely from day to day, ask:

What is the total amount of time you spent in a typical week over the last 3 months doing at least moderate physical activities inside your home?

|__|__|:|__|__| *Hours: minutes per week (8800 = refused, 9900 = don't know)*

PART 3: TRAVEL, RECREATION, SPORT, AND LEISURE-TIME PHYSICAL ACTIVITY

LEISL

57. Think about the times that you spent walking to get from place to place, to do errands, and any other walking that you might do solely for recreation, leisure, exercise, or sport. This includes time spent power walking and playing golf.

[if person works ≥4 hours per week say next sentence:]

It also includes walking to and from work and during work breaks such as at lunchtime, but not walking you did as part of your work.

LEISWLK

In a typical week during the last 3 months, on how many days did you <u>walk</u> for at least 10 minutes at a time?

|__| *Days per week* *[If **0**, Go to Question 60]*

8 Refused *[Go to Question 60]*

9 Don't Know/Not Sure *[Go to Question 60]*

LEISWLKU

58. On the days when you walked, how much time did you usually spend walking in a single day?

LEISWLKT

|__|__|:|__|__| *Hours: minutes per day* *(8800 = refused, 9900 = don't know)*

IF NECESSARY prompt with: An average time per day is being sought.

INTERVIEWER CLARIFICATION
If the respondent can't answer because the pattern of time spent varies widely from day to day, ask:
What is the total amount of time you spent walking <u>in a typical week</u> over the last 3 months?
|__|__|:|__|__| *Hours: minutes per week* *If answer is 'refused' or 'don't know' go to question 60.*
(8800 = refused, 9900 = don't know)

WLKPACE

59. What is your usual walking pace outdoors?

READ OUT

1 Slow

2 Normal or average

3 Brisk pace

4 Very brisk, striding

8 Refused (DO NOT READ)

9 Don't know/Not sure (DO NOT READ)

LEISVIG

60. Now, think about <u>vigorous</u> physical activities that you did to get from place to place, to do errands, or solely for recreation, sport, exercise and leisure. Vigorous activities take <u>hard physical</u> effort and make you breathe <u>much</u> harder than normal. Some examples are aerobics, running, fast bicycling, or fast swimming. Do not include walking. Include only those physical activities that you did for at least 10 minutes at a time.

In a typical week during the last 3 months, on how many days did you do <u>vigorous</u> physical activities <u>in your leisure time</u>?

|__| *Days per week* *[If **0**, Go to Question 62]*

8 Refused (DO NOT READ) [Go to Question 62]

9 Don't Know/Not Sure (DO NOT READ) [Go to Question 62]

LEISVIGU

61. On the days when you did vigorous activities in your leisure time, how much time did you usually spend doing them in a single day?

LEISVIGT

|__|__|:|__|__| *Hours: minutes per day* *(8800 = refused, 9900 = don't know)*

IF NECESSARY prompt with: An average time per day is being sought.

INTERVIEWER CLARIFICATION
If the respondent can't answer because the pattern of time spent varies widely from day to day, ask:

What is the total amount of time you spent in a typical week over the last 3 months doing vigorous physical activities in your leisure time?

|__|__|:|__|__| *Hours: minutes per week* *(8800 = refused, 9900 = don't know)*

LEISMOD

62. Now think about activities which take moderate physical effort that you did in your leisure time or to get from one place to another. Some examples are bicycling at an easy pace, swimming at an easy pace, and doubles tennis. Do not include walking. Again, include only those moderate activities that you did for at least 10 minutes at a time.

In a typical week during the last 3 months, on how many days did you do <u>moderate</u> physical activities <u>in your leisure time</u>?

INTERVIEWER CLARIFICATION:
Moderate physical activities make you breathe somewhat harder than normal.

|__| *Days per week* *[If **0**, Go to Question 64]*

8 Refused (DO NOT READ) [Go to question 64]

9 Don't Know/Not Sure (DO NOT READ) [Go to question 64]

LEISMODU

63. On the days when you did moderate activities in your leisure time, how much time did you usually spend doing them in a single day?

LEISMODT

|__|__|**:**|__|__| *Hours: minutes per day* *(8800 = refused, 9900 = don't know)*

IF NECESSARY prompt with: An average time per day is being sought.

INTERVIEWER CLARIFICATION:
If the respondent can't answer because the pattern of time spent varies widely from day to day, ask:

What is the total amount of time you spent in a typical week over the last 3 months doing moderate physical activities in your leisure time?

|__|__|**:**|__|__| *Hours: minutes per week* *(8800 = refused, 9900 = don't know)*

PART 4: TIME SPENT SITTING

TELEWRK

The last questions in this section are about the time you spend watching television.

64. In a typical week during the last 3 months, how much time did you usually spend <u>watching television</u> on a <u>working day or weekday</u>?

INTERVIEWER CLARIFICATION:
Watching television includes watching videos and DVDs.
Working day or weekday is the whole day including the evenings.

|__|__|:|__|__| *Hours: minutes per day (2100 = None 2200 = Refused, 2300 = Don't know)*

TELEWRKN

65. In a typical week during the last 3 months, how much time did you usually spend <u>watching television</u> on a <u>non-working day or weekend day</u>?

INTERVIEWER CLARIFICATION:
Watching television includes watching videos and DVDs.
Non working day or weekend day is the whole day including the evenings.

|__|__|:|__|__| *Hours: minutes per day (2100 = None 2200 = Refused, 2300 = Don't know)*

SECTION 7 HEALTH

HA

66. The next questions are about your health and medical procedures you may have had.

Has a doctor or nurse ever told you that you had a <u>heart attack</u>? (also called a <u>myocardial infarction)</u>

INTERVIEWER CLARIFICATION: A heart attack is also a coronary, coronary occlusion or myocardial infarction (irreversible damage) to part of the heart. A heart attack is not heart failure, pulmonary embolism, heart aneurysm, or cardiomyopathy.

1	*Yes*		
2	*No*		*Go to question 70*
8	*Refused*	*(DO NOT READ)*	*Go to question 70*
9	*Don't Know/Not Sure*	*(DO NOT READ)*	*Go to question 70*

HAUNT

67. When did your <u>last</u> heart attack occur?

HAAGE/HAYR/HAYRS/HAMTHS

INTERVIEWER INSTRUCTION:
If year is current year probe for months ago.

Age |__|__| or Year |__|__||__|__| or Years ago |__|__| or Months ago |__|__|

[Refused, age = 88, Don't know, age = 99]

HAHSP

68. Were you hospitalised for at least one night?

1	*Yes*		
2	*No*		*Go to question 70*
8	*Refused*	*(DO NOT READ)*	*Go to question 70*
9	*Don't Know/Not Sure*	*(DO NOT READ)*	*Go to question 70*

HAHSP2

69. What was the name of the hospital?

____

BYP

70. Have you ever had heart or coronary bypass surgery?

INTERVIEWER CLARIFICATION: This is a procedure to improve blood flow to the heart muscle when the heart's arteries are narrowed or blocked. Veins from your leg or arteries from your wrist or within your chest are used to bypass blockages in the heart's arteries.

1	*Yes*		
2	*No*		*Go to question 73*
8	*Refused*	*(DO NOT READ)*	*Go to question 73*
9	*Don't Know/Not Sure*	*(DO NOT READ)*	*Go to question 73*

BYPUNT

71. When did this <u>last</u> happen?

BYPAGE/BYPYR/BYPYRS/BYPMTHS

INTERVIEWER INSTRUCTION:
If year is current year probe for months ago.

Age |__|__| or Year |__|__||__|__| or Years ago |__|__| or Months ago |__|__|

[Refused, age = 88, Don't know, age = 99]

BYPHSP

72. What was the name of the hospital where it was performed?

____

COR

73. The next question is about coronary angioplasty and coronary stent.

Angioplasty is a procedure to improve blood flow to the heart muscle when the heart's arteries are narrowed or blocked.

A stent is a metal tube, which may be inserted at the same time to help arteries stay unblocked.

Have you ever had a coronary angioplasty or coronary stent?

INTERVIEWER CLARIFICATION: Angiography or an angiogram is a procedure in which a needle is inserted into your groin and a special tube passed through to the heart arteries where dye is released so that the doctor can use an Xray to look for blockages. Angioplasty is done during an angiogram when the doctor has found a blockage, a deflated balloon is passed through to where the blockage is, then inflated to open up the blocked artery.

1	*Yes*		
2	*No*		*Go to question 76*
8	*Refused*	*(DO NOT READ)*	*Go to question 76*
9	*Don't Know/Not Sure*	*(DO NOT READ)*	*Go to question 76*

CORUNT

74. When did this last happen?

CORAGE/CORYR/CORYRS/CORMTHS

INTERVIEWER INSTRUCTION:
If year is current year probe for months ago.

Age |__|__| or Year |__|__||__|__| or Years ago |__|__| or Months ago |__|__|

[Refused, age = 88, Don't know, age = 99]

CORHSP

75. What was the name of the hospital where it was performed?

____

STK

76. Has a doctor or nurse ever told you that you had a stroke?

1 Yes

2 No *Go to question 80*

8 Refused *(DO NOT READ)* *Go to question 80*

9 Don't Know/Not Sure *(DO NOT READ)* *Go to question 80*

INTERVIEWER CLARIFICATION: A stroke is also known as / caused by and embolism to the brain, a clot to / in the brain, cerebrovascular accident (CVA) , cerebral haemorrhage. Bleeding in to the brain or brain aneurysm (only if it bursts and bleeds)

This is irreversible damage to part of the brain, resulting in permanent neurological deficits, such as weakness, numbness, or speech impairment.

A stroke is not a TIA (transient ischaemic attack), or mini stroke

STKUNT

77. When did your last stroke occur?

STKAGE/ATKYR/ATKYRS/STKMTHS

INTERVIEWER INSTRUCTION:
If year is current year probe for months ago.

Age |__|__| or Year |__|__||__|__| or Years ago |__|__| or Months ago |__|__|

[Refused, age = 88, Don't know, age = 99]

STKHSP

78. Were you hospitalised for at least one night?

1 Yes

2 No *Go to question 80*

8 Refused *(DO NOT READ)* *Go to question 80*

9 Don't Know/Not Sure *(DO NOT READ)* *Go to question 80*

STHSP2

79. What was the name of the hospital?

____

ANG

80. Has a doctor ever told you that you have 'angina'?

INTERVIEWER CLARIFICATION: Angina is a temporary chest pain or discomfort, resulting from a reduced blood supply to the heart muscle. It usually occurs during exercise or effort, or in response to emotion, and is usually relieved by rest or medication.

1	*Yes*		
2	*No*		*Go to question 82*
8	*Refused*	*(DO NOT READ)*	*Go to question 82*
9	*Don't Know/Not sure*	*(DO NOT READ)*	*Go to question 82*

ANGUNT

81. When were you first told you had angina?

ANGAGE/ ANGYR/ ANGYRS/ ANGMTHS

INTERVIEWER INSTRUCTION:
If year is current year probe for months ago.

Age |__|__| or Year |__|__||__|__| or Years ago |__|__| or Months ago |__|__|

[Refused, age = 88, Don't know, age = 99]

HBCURR

The next questions are about heartburn, which is a burning pain behind the breastbone in your chest. It often occurs after eating.

82. Have you had heartburn in the last 12 months?

1	*Yes*		
2	*No*		*Go to question 85*
8	*Refused*	*(DO NOT READ)*	*Go to question 85*
9	*Don't Know/Not Sure*	*(DO NOT READ)*	*Go to question 85*

HBFREQ

83. In the last 12 months, how often did you get heartburn? Would you say …

READ OUT CHOICES

**1**	**Daily**	
**2**	**Several days a week**	
**3**	**About once a week**	
**4**	**About once a fortnight**	*Go to question 85*
**5**	**About once a month**	*Go to question 85*

6 Less than once a month *Go to question 85*

8 Refused (DO NOT READ) Go to question 85

9 Don't Know/Not Sure (DO NOT READ) Go to question 85

HBLMT

84. When you are suffering from heartburn, to what extent does it limit you in any of your activities? Would you say…

READ OUT CHOICES

1 A great extent *Go to question 89*

2 A moderate extent *Go to question 89*

3 Only slightly *Go to question 89*

4 Not at all *Go to question 89*

8 Refused (DO NOT READ) Go to question 89

9 Don't Know/Not Sure (DO NOT READ) Go to question 89

HBPST

85. In the past, have you ever had heartburn at least once a week?

1 Yes

2 No Go to question 90

8 Refused (DO NOT READ) Go to question 90

9 Don't Know/Not Sure (DO NOT READ) Go to question 90

HBL

86. When did you last have heartburn at least once a week?

HBLAGE/HBLYR/HBLYRS/HBLMTHS

INTERVIEWER INSTRUCTION:
If year is current year probe for months ago.

Age |__|__| *or Year* |__|__||__|__| *or Years ago* |__|__| *or Months ago* |__|__|

Only during pregnancy **|__|**

[Refused, age = 88, Don't know, age = 99]

HBOFT

87. When you last had heartburn at least once a week, how often did you get it? Would you say …

READ OUT CHOICES

1 Daily

2 Several days a week

3 About once a week

8 Refused (DO NOT READ) skip to question 89

9 Don't Know/Not Sure (DO NOT READ) skip to question 89

HBSUFF

88. When you suffered from heartburn, to what extent did it limit you in any of your activities? Would you say…

READ OUT CHOICES

1 A great extent

2 A moderate extent

3 Only slightly

4 Not at all

8 Refused (DO NOT READ)

9 Don't Know/Not Sure (DO NOT READ)

HBUNT

89. When did you first have heartburn at least once a week?

HBAGE/HBYR/HBYRS/HBMTHS

INTERVIEWER INSTRUCTION:
If year is current year probe for months ago.

Age |__|__| or Year |__|__||__|__| or Years ago |__|__| or Months ago |__|__|

Only during pregnancy **|__|**

[Refused, age = 88, Don't know, age = 99]

RFCURR

The next questions are about acid reflux or regurgitation, which is a bitter or sour taste from acid or bile coming up into your mouth or throat.

90. Have you had acid reflux in the last 12 months?

1 Yes

2 No Go to question 93

8 Refused (DO NOT READ) Go to question 93

9 Don't Know/Not Sure (DO NOT READ) Go to question 93

RFFREQ

91. In the last 12 months, how often did you get acid reflux? Would you say …

READ OUT CHOICES

1 Daily

2 Several days a week

3 About once a week

4 About once a fortnight *Go to question 93*

5 About once a month *Go to question 93*

6 Less than once a month *Go to question 93*

8 Refused (DO NOT READ) Go to question 93

9 Don't Know/Not Sure (DO NOT READ) Go to question 93

RFLMT

92. When you are suffering from acid reflux, to what extent does it limit you in any of your activities? Would you say…

READ OUT CHOICES

1 A great extent *Go to question 97*

2 A moderate extent *Go to question 97*

3 Only slightly *Go to question 97*

4 Not at all *Go to question 97*

8 Refused (DO NOT READ) Go to question 97

9 Don't Know/Not Sure (DO NOT READ) Go to question 97

RFPST

93. In the past, have you ever had acid reflux at least once a week?

1 Yes

2 No Go to question 97

8 Refused (DO NOT READ) Go to question 97

9 Don't Know/Not Sure (DO NOT READ) Go to question 97

RFL

94. When did you <u>last</u> have acid reflux at least once a week?

RFLAGE/RFLYR/RFLYRS/RFLMTHS

INTERVIEWER INSTRUCTION:
If year is current year probe for months ago.

Age |__|__| or Year |__|__||__|__| or Years ago |__|__| or Months ago |__|__|

Only during pregnancy **|__|**

[Refused, age = 88, Don't know, age = 99]

RFOFT

95. When you last had acid reflux at least once a week, how often did you get it? Would you say …

READ OUT CHOICES

1 Daily

2 Several days a week

3 About once a week

8 Refused (DO NOT READ) skip to question 97

9 Don't Know/Not Sure (DO NOT READ) skip to question 97

RFSUFF

96. When you suffered from acid reflux, to what extent did it limit you in any of your activities? Would you say…

READ OUT CHOICES

1 A great extent

2 A moderate extent

3 Only slightly

4 Not at all

8 Refused (DO NOT READ)

9 Don't Know/Not Sure (DO NOT READ)

RFUNT

97. When did you first have acid reflux at least once a week?

RFAGE/RFYR/RFYRS/RFMTHS

INTERVIEWER INSTRUCTION:
If year is current year probe for months ago.

Age |__|__| or Year |__|__||__|__| or Years ago |__|__| or Months ago |__|__|

Only during pregnancy |__|

[Refused, age = 88, Don't know, age = 99]

HBRFDR1

98. In the last 12 months, have you taken any prescription medication for heartburn or acid reflux?

INTERVIEWER CLARIFICATION:
Prescription medication is medication prescribed by a doctor. Some commonly prescribed medications are Losec, Somac, Nexium, Pariet, Zoton, Acimax, Zantac and Tazac.

1 Yes

2 No

8 Refused (DO NOT READ)

9 Don't Know/Not Sure (DO NOT READ)

HBRFDR2

99. In the last 12 months, have you taken any over-the-counter medication for heartburn or acid reflux?

INTERVIEWER CLARIFICATION:
Over-the-counter medication is medication purchased without a prescription. Some common over the counter ones are Mylanta, Gaviscon, Gastrogel and Zantac.

1 Yes

2 No

8 Refused (DO NOT READ)

9 Don't Know/Not Sure (DO NOT READ)

INTERVIEWER INSTRUCTION:
Only ask the <u>next two questions</u> if 'yes' was answered to <u>at least one of the two previous questions</u>.

HBRFDRF

100. How often do you usually take medication for heartburn or acid reflux?

Would you say …

INTERVIEWER CLARIFICATION:
This question is about over-the-counter and prescription medication.

1 Daily

2 Several days a week

3 About once a week

4 About once a fortnight

5 About once a month

6 Less than once a month

8 Refused (DO NOT READ)

9 Don't Know/Not Sure (DO NOT READ)

HBRFDRY

101. For how many years have you taken these medicines?

1 Less than one year

2 1 to 2 years

3 2 to 4 years

4 5-9 years

5 10 or more years

8 Refused (DO NOT READ)

9 Don't Know/Not Sure (DO NOT READ)

BE

102. Has a doctor ever told you that you have Barrett's oesophagus?

INTERVIEWER CLARIFICATION:
Barrett's oesophagus is a condition that affects the lining of the oesophagus. It can only be diagnosed by directly inspecting the oesophagus with an endoscope. The procedure is usually done under a light sedative as a day-case patient in a specialised endoscopy unit or hospital.

1	*Yes*		
2	*No*		*Go to question 105*
8	*Refused*	*(DO NOT READ)*	*Go to question 105*
9	*Don't Know/Not Sure*	*(DO NOT READ)*	*Go to question 105*

BEUNT

103. When were you first told you had Barrett's oesophagus?

BEAGE/BEYR/BEYRS/BEMTHS

INTERVIEWER INSTRUCTION:
If year is current year probe for months ago.

Age |__|__| or Year |__|__||__|__| or Years ago |__|__| or Months ago |__|__|

[Refused, age = 88, Don't know, age = 99]

BEDOC

104. What is the name of the doctor who first told you had Barrett's oesophagus?

____

DIAB

105. Has a doctor ever told you that you have diabetes?

INTERVIEWER CLARIFICATION: Diabetes is a condition where there is too much glucose (sugar) in the blood.

1	*Yes*		
2	*No*		*Go to question 107*
8	*Refused*	*(DO NOT READ)*	*Go to question 107*
9	*Don't Know/Not sure*	*(DO NOT READ)*	*Go to question 107*

DIABUNT

106. When were you first told you had diabetes?

DIABAGE/DIABYR/DIABYRS/DIABMTHS

INTERVIEWER INSTRUCTION:
If year is current year probe for months ago.

Age |__|__| or Year |__|__||__|__| or Years ago |__|__| or Months ago |__|__|

[Refused, age = 88, Don't know, age = 99]

ACNE

107. Have you ever had acne?

INTERVIEWER CLARIFICATION
Pimples are also considered to be Acne.

1	*Yes*		
2	*No*		*Go to question 115*
8	*Refused*	*(DO NOT READ)*	*Go to question 115*
9	*Don't Know/Not sure*	*(DO NOT READ)*	*Go to question 115*

ACNEAGE

108. At what age did you develop acne?

ACNEAGE/ACNEYR

Age |__|__| or Year |__|__||__|__|

[Refused, age = 88, Don't know, age = 99]

ACNETRT

109. Did you require treatment for your acne?

1	*Yes*		
2	*No*		*Go to question 113*
8	*Refused*	*(DO NOT READ)*	*Go to question 113*
9	*Don't Know/Not sure*	*(DO NOT READ)*	*Go to question 113*

ACNEOTC

110. Did you seek treatment…..

Over the counter?

1	*Yes*	
2	*No*	
8	*Refused*	*(DO NOT READ)*
9	*Don't Know/Not sure*	*(DO NOT READ)*

ACNEDOC

111. From your doctor?

1 Yes

2 No

8 Refused (DO NOT READ)

9 Don't Know/Not sure (DO NOT READ)

ACNEDER

112. From a dermatologist?

1 Yes

2 No

8 Refused (DO NOT READ)

9 Don't Know/Not sure (DO NOT READ)

ACNESCR2

113. Do you have any scarring due to acne?

1 Yes

2 No

8 Refused (DO NOT READ)

9 Don't Know/Not sure (DO NOT READ)

ACNEBDY

114. Whereabouts on your body did you get acne?

Enter all that apply.

READ OUT CHOICES

1 Face

2 Neck / Shoulders

3 Back

4 Chest

8 Refused (DO NOT READ)

9 Don't Know/Not sure (DO NOT READ)

HVS

115. Has a doctor ever told you that you have hives?

1	*Yes*		
2	*No*		*go to question 118*
8	*Refused*	*(DO NOT READ)*	*go to question 118*
9	*Don't Know/Not sure*	*(DO NOT READ)*	*go to question 118*

HVSUNT

116. When were you first told you had hives?

HVSAGE/HVSYR/HVSYRS/HVSMTHS

INTERVIEWER INSTRUCTION:
If year is current year probe for months ago.

Age |__|__| or Year |__|__||__|__| or Years ago |__|__| or Months ago |__|__|

[Refused, age = 88, Don't know, age = 99]

HVSMED

117. Did/do you take any medication for hives?

1	*Yes*	
2	*No*	
8	*Refused*	*(DO NOT READ)*
9	*Don't Know/Not sure*	*(DO NOT READ)*

HYFV

118. Has a doctor ever told you that you have hay fever?

1	*Yes*		
2	*No*		*go to question 121*
8	*Refused*	*(DO NOT READ)*	*go to question 121*
9	*Don't Know/Not sure*	*(DO NOT READ)*	*go to question 121*

HYFVUNT

119. When were you first told you had hay fever?

HYFVAGE/HYFVYR/HYFVYRS/HYFVMTHS

INTERVIEWER INSTRUCTION:
If year is current year probe for months ago.

Age |__|__| or Year |__|__||__|__| or Years ago |__|__| or Months ago |__|__|

[Refused, age = 88, Don't know, age = 99]

HYFVMED

120. Did/do you take any medication for hay fever?

1 Yes

2 No

8 Refused (DO NOT READ)

9 Don't Know/Not sure (DO NOT READ)

ECZM

121. Has a doctor ever told you that you have eczema / dermatitis?

1 Yes

2 No *go to question 124*

8 Refused (DO NOT READ) *go to question 124*

9 Don't Know/Not sure (DO NOT READ) *go to question 124*

ECZMUNT

122. When were you first told you had eczema / dermatitis?

ECZMAGE/ECZMYR/ECZMYRS/ECZMMTHS

INTERVIEWER INSTRUCTION:
If year is current year probe for months ago.

Age |__|__| or Year |__|__||__|__| or Years ago |__|__| or Months ago |__|__|

[Refused, age = 88, Don't know, age = 99]

ECZMMED

123. Did/do you take any medication for eczema / dermatitis?

1 Yes

2 No

8 Refused (DO NOT READ)

9 Don't Know/Not sure (DO NOT READ)

FDAL

124. Has a doctor ever told you that you have food allergy?

1	*Yes*		
2	*No*		*go to question 127*
8	*Refused*	*(DO NOT READ)*	*go to question 127*
9	*Don't Know/Not sure*	*(DO NOT READ)*	*go to question 127*

FDALUNT

125. When were you first told you had food allergy?

FDALAGE/FDALYR/FDALYRS/FDALMTHS

INTERVIEWER INSTRUCTION:
If year is current year probe for months ago.

Age |__|__| or Year |__|__||__|__| or Years ago |__|__| or Months ago |__|__|

[Refused, age = 88, Don't know, age = 99]

FDALMED

126. Did/do you take any medication for food allergy?

1	*Yes*	
2	*No*	
8	*Refused*	*(DO NOT READ)*
9	*Don't Know/Not sure*	*(DO NOT READ)*

DRAL

127. Has a doctor ever told you that you have allergy to drugs?

1	*Yes*		
2	*No*		*go to question 130*
8	*Refused*	*(DO NOT READ)*	*go to question 130*
9	*Don't Know/Not sure*	*(DO NOT READ)*	*go to question 130*

DRALUNT

128. When were you first told you had allergy to drugs?

DRALAGE/DRALYR/DRALYRS/DRALMTHS

INTERVIEWER INSTRUCTION:
If year is current year probe for months ago.

Age |__|__| or Year |__|__||__|__| or Years ago |__|__| or Months ago |__|__|

[Refused, age = 88, Don't know, age = 99]

DRALMED

129. Did/do you take any medication for allergy to drugs?

1 Yes

2 No

8 Refused (DO NOT READ)

9 Don't Know/Not sure (DO NOT READ)

ASTH

130. Has a doctor ever told you that you have asthma or wheezing?

1 Yes

2 No *go to question 133*

8 Refused (DO NOT READ) *go to question 133*

9 Don't Know/Not sure (DO NOT READ) *go to question 133*

ASTHUNT

131. When were you first told you had asthma or wheezing?

ASTHAGE/ASTHYR/ASTHYRS/ASTHMTHS

INTERVIEWER INSTRUCTION:
If year is current year probe for months ago.

Age |__|__| or Year |__|__||__|__| or Years ago |__|__| or Months ago |__|__|

[Refused, age = 88, Don't know, age = 99]

ASTHMED

132. Did/do you take any medication for asthma or wheezing?

1 Yes

2 No

8 Refused (DO NOT READ)

9 Don't Know/Not sure (DO NOT READ)

ARTH

133. Has a doctor ever told you that you have arthritis?

1 Yes

2 No

8 Refused (DO NOT READ)

9 Don't Know/Not sure (DO NOT READ)

HIP

134. Have you ever had a hip replacement?

1	*Yes*		
2	*No*		*Go to question 137*
8	*Refused*	*(DO NOT READ)*	*Go to question 137*
9	*Don't Know*	*(DO NOT READ)*	*Go to question 137*

HIPUNT

135. When did you have your first hip replacement?

HIPAGE/HIPYR/HIPYRS/HIPMTHS

INTERVIEWER INSTRUCTION:
If year is current year probe for months ago.

Age |__|__| or Year |__|__||__|__| or Years ago |__|__| or Months ago |__|__|

[Refused, age = 88, Don't know, age = 99]

HIPHOSP

136. What was the name of the hospital where this was done?

____

KNEE

137. Have you ever had a knee replacement?

1	*Yes*		
2	*No*		*Go to question 140*
8	*Refused*	*(DO NOT READ)*	*Go to question 140*
9	*Don't Know/Not sure*	*(DO NOT READ)*	*Go to question 140*

KNEEUNT

138. When did you have your first knee replacement?

KNEEAGE/KNEEYR/KNEEYRS/KNEEMTHS

INTERVIEWER INSTRUCTION:
If year is current year probe for months ago.

Age |__|__| or Year |__|__||__|__| or Years ago |__|__| or Months ago |__|__|

[Refused, age = 88, Don't know, age = 99]

KNEEHOSP

139. What was the name of the hospital that this was done?

____

FRACBONE

140. Have you broken or fractured a bone since you were 50 years old?

1	*Yes*		
2	*No*		*Go to question 147*
8	*Refused*	*(DO NOT READ)*	*Go to question 147*
9	*Don't Know/Not sure*	*(DO NOT READ)*	*Go to question 147*

FRACHIP

141. Since you were 50, have you ever broken or fractured your hip?

1	*Yes*		
2	*No*		*Go to question 143*
8	*Refused*	*(DO NOT READ)*	*Go to question 143*
9	*Don't Know/Not Sure*	*(DO NOT READ)*	*Go to question 143*

FRACHIPC

142. Did that fracture occur as a result of a fall from

INTERVIEWER CLARIFICATION: If person says that they have had more than one, tell them to think about the last one.

A break or fracture caused by a sporting accident, even if from a standing height i.e. skiing, should be recorded as a severe trauma.

READ OPTIONS

1	Standing height or less	
2	Because of a harder fall or	
3	From a car accident or other severe trauma	
8	*Refused*	*(DO NOT READ)*
9	*Don't Know/Not sure*	*(DO NOT READ)*

FRACWST

143. Since you were 50, have you ever broken or fractured your wrist?

1	*Yes*		
2	*No*		*Go to question 145*
8	*Refused*	*(DO NOT READ)*	*Go to question 145*
9	*Don't Know/Not sure*	*(DO NOT READ)*	*Go to question 145*

FRACWSTC

144. Did that fracture occur as a result of a fall from

INTERVIEWER CLARIFICATION: If person says that they have had more than one, tell them to think about the last one.

A break or fracture caused by a sporting accident, even if from a standing height i.e. skiing, should be recorded as a severe trauma.

READ OPTIONS

1 Standing height or less

2 Because of a harder fall or

3 From a car accident or other severe trauma

8 Refused (DO NOT READ)

9 Don't Know/Not sure (DO NOT READ)

FRACSHD

145. Since you were 50, have you ever broken or fractured your shoulder?

1	*Yes*		
2	*No*		*Go to question 147*
8	*Refused*	*(DO NOT READ)*	*Go to question 147*
9	*Don't Know/Not sure*	*(DO NOT READ)*	*Go to question 147*

FRACSHDC

146. Did that fracture occur as a result of a fall from

INTERVIEWER CLARIFICATION: If person says that they have had more than one, tell them to think about the last one.

A break or fracture caused by a sporting accident, even if from a standing height i.e. skiing, should be recorded as a severe trauma.

READ OPTIONS

1 Standing height or less

2 Because of a harder fall or

3 From a car accident or other severe trauma

8 Refused (DO NOT READ)

9 Don't Know/Not sure (DO NOT READ)

GSCP

147. Now I will ask you about a procedure used by a doctor to look at the inside of the oesophagus and the stomach. It is called a gastroscopy or sometimes, an upper GI endoscopy.

In a gastroscopy, a tube is passed throught the mouth into the oesophagus and the stomach.

INTERVIEWER CLARIFICATION:
The procedure is usually done under a light sedative as a day-case patient in a specialised endoscopy unit or hospital.

Have you ever had a gastroscopy?

1	*Yes*		
2	*No*		*Go to question 150*
8	*Refused*	*(DO NOT READ)*	*Go to question 150*
9	*Don't know/Not sure*	*(DO NOT READ)*	*Go to question 150*

GSCPUNT

148. When was your <u>first</u> gastroscopy?

GSCPAGE/GSCPYR/GSCPYRS/GSCPMTHS

INTERVIEWER INSTRUCTION:
If year is current year probe for months ago.

Age |__|__| or Year |__|__||__|__| or Years ago |__|__| or Months ago |__|__|

[Refused, age = 88, Don't know, age = 99]

GSCPHOSP

149. What was the name of the Doctor who performed this procedure?

INTERVIEWER CLARIFICATION:

If participant is not sure of name of Doctor, probe for name of hospital or day procedure centre.

____

COLO

150. The next procedures I want to ask you about are used to examine the bowel.

In a colonoscopy, a tube is passed through the entire large bowel. In preparing for the colonoscopy, you may have had an enema or taken a large quantity of liquid preparation to empty the bowels.

INTERVIEWER CLARIFICATION: A colonoscopy is usually performed with anaesthesia.

Have you ever had a colonoscopy?

1	*Yes*	
2	*No*	*Go to question 152*

8	*Refused*	*(DO NOT READ)*	*Go to question 152*
9	*Don't Know/Not sure*	*(DO NOT READ)*	*Go to question 152*

COLUNT

151. When was your last colonoscopy?

COLAGE/COLYR/COLYRS/COLMTHS

INTERVIEWER INSTRUCTION:
If year is current year probe for months ago.

Age |__|__| or Year |__|__||__|__| or Years ago |__|__| or Months ago |__|__|

[Refused, age = 88, Don't know, age = 99]

SIGM

152. In a sigmoidoscopy, a tube is passed into the lower bowel and rectum. The examination is usually done in a doctor's office without anaesthesia.

Have you ever had a sigmoidoscopy?

1	*Yes*		
2	*No*		*Go to question 154*
8	*Refused*	*(DO NOT READ)*	*Go to question 154*
9	*Don't know/Not sure*	*(DO NOT READ)*	*Go to question 154*

SIGUNT

153. When was your last sigmoidoscopy?

SIGAGE/SIGYR/SIGYRS/SIGMTHS

INTERVIEWER INSTRUCTION:
If year is current year probe for months ago.

Age |__|__| or Year |__|__||__|__| or Years ago |__|__| or Months ago |__|__|

[Refused, age = 88, Don't know, age = 99]

FOBT

154. A faecal occult blood test or FOBT is a test in which a sample of faeces is smeared onto specially treated cards. It is also called a stool smear test or haemoccult test.

It is a test usually done at home.

Have you ever had a faecal occult blood test?

INTERVIEWER CLARIFICATION: This does not include a stool sample placed in a specimen jar.

1 Yes

2 No *Go to NEXT SECTION – 'Feeling'*

8 Refused *Go to NEXT SECTION – 'Feeling'*

9 Don't know/Not sure *Go to NEXT SECTION – 'Feeling'*

FOBTUNT

155. When was your <u>last</u> faecal occult blood test?

FOBTAGE/AOBTYR/FOBTYRS/FOBTMTHS

INTERVIEWER INSTRUCTION:
If year is current year probe for months ago.

Age |__|__| or Year |__|__||__|__| or Years ago |__|__| or Months ago |__|__|

[Refused, age = 88, Don't know, age = 99]

SECTION 8 PAUSE TO ASK RESPONDENT HOW HE/SHE IS FEELING

FEELING

Interviewer to say something like:

How are you feeling right now? Would you like to have any sort of a break for a minute?

If the respondent asks "How much longer will it take / do we have to go?" Say:

We are well past the half way point of the interview, so there's really not long to go – just a couple more sections. We really appreciate all of the information you have given us so far.

SECTION 9 FAMILY HISTORY

The next questions are about cancer, heart disease and stroke in your parents, brothers, and sisters. I want to know this information for relatives who are alive and for any that have died.

MUMCRC

156. The first questions are about your mother.

[if the respondent tells you that he/she is adopted, do not ask the question about bowel cancer in the mother, but record an answer of 7 for this question (adopted).]

INTERVIEWER INSTRUCTION:
DO NOT ASK the respondent if their mother is alive or dead.

If respondent has told you that their mother is alive ask:

Has your mother ever had bowel cancer?

If respondent has told you that their mother has died ask:

Did your mother ever have bowel cancer?

INTERVIEWER CLARIFICATION: Bowel cancer is sometimes called colon cancer or rectal cancer or colorectal cancer.

1	*Yes*		
2	*No*		*Go to question 158*
7	*Participant is adopted*		*Go to NEXT SECTION – question 190 if participant is MALE, question 195 if participant if FEMALE*
8	*Refused*	*(DO NOT READ)*	*Go to question 158*
9	*Don't know/Not sure*	*(DO NOT READ)*	*Go to question 158*

MUMCRCDX

157. How old was she when it was first diagnosed?

|__|__| *years old (Don't know = 99, if > 98 enter 98)*

MUMBC

158. *If respondent has told you that their mother is alive ask:*
Has she ever had breast cancer?

If respondent has told you that their mother has died ask:
Did she ever have breast cancer?

1	*Yes*		
2	*No*		*Go to question 160*
8	*Refused*	*(DO NOT READ)*	*Go to question 160*
9	*Don't know/Not sure*	*(DO NOT READ)*	*Go to question 160*

MUMBCDX

159. How old was she when it was first diagnosed?

|__|__| *years old (Don't know = 99, if > 98 enter 98)*

MUMHA

160. *If respondent has told you that their mother is alive ask:*
Has she ever had a '<u>heart attack</u>'? (also called a myocardial infarction)

If respondent has told you that their mother has died ask:
Did she ever have a '<u>heart attack</u>'? (also called a myocardial infarction)

1	*Yes*		
2	*No*		*Go to question 162*
8	*Refused*	*(DO NOT READ)*	*Go to question 162*
9	*Don't know/Not sure*	*(DO NOT READ)*	*Go to question 162*

MUMHADX

161. How old was she when she had her first heart attack?

|__|__| *years old (Don't know = 99, if > 98 enter 98)*

MUMSTK

162. *If respondent has told you that their mother is alive ask:*
Has she ever had a stroke?

If respondent has told you that their mother has died ask:
Did she ever have a stroke?

1	*Yes*		
2	*No*		*Go to question 164*
8	*Refused*	*(DO NOT READ)*	*Go to question 164*
9	*Don't know/Not sure*	*(DO NOT READ)*	*Go to question 164*

MUMSTKDX

163. How old was she when she had her first stroke?

|__|__| *years old (Don't know = 99, if > 98 enter 98)*

DADCRC

164. The next questions are about your father

If respondent has told you that their father is alive ask:
Has he ever had bowel cancer?

If respondent has told you that their father has died ask:
Did he ever have bowel cancer?

1	*Yes*		
2	*No*		*Go to question 166*
8	*Refused*	*(DO NOT READ)*	*Go to question 166*
9	*Don't know/Not sure*	*(DO NOT READ)*	*Go to question 166*

DADCRCDX

165. How old was he when it was first diagnosed?

|__|__| *years old (Don't know = 99, if > 98 enter 98)*

DADPC

166. *If respondent has told you that their father is alive ask:*
Has he ever had prostate cancer?

If respondent has told you that their father has died ask:
Did he ever have prostate cancer?

1	*Yes*		
2	*No*		*Go to question 168*
8	*Refused*	*(DO NOT READ)*	*Go to question 168*
9	*Don't know/Not sure*	*(DO NOT READ)*	*Go to question 168*

DADPCDX

167. How old was he when it was first diagnosed?

|__|__| *years old (Don't know = 99, if > 98 enter 98)*

DADHA

168. *If respondent has told you that their father is alive ask:*
Has he ever had a '<u>heart attack</u>'? (also called a myocardial infarction)

If respondent has told you that their father has died ask:
Did he ever have a '<u>heart attack</u>'? (also called a myocardial infarction)

1	*Yes*		
2	*No*		*Go to question 170*
8	*Refused*	*(DO NOT READ)*	*Go to question 170*
9	*Don't know/Not sure*	*(DO NOT READ)*	*Go to question 170*

DADHADX

169. How old was he when he had his first heart attack?

|__|__| *years old (Don't know = 99, if > 98 enter 98)*

DADSTK

170. *If respondent has told you that their father is alive, ask:*
Has he ever had a stroke?

If respondent has told you that their father has died, ask:
Did he ever have a stroke?

1	*Yes*		
2	*No*		*Go to question 172*
8	*Refused*	*(DO NOT READ)*	*Go to question 172*
9	*Don't know/Not sure*	*(DO NOT READ)*	*Go to question 172*

DADSTKDX

171. How old was he when he had his first stroke?

|__|__| *years old (Don't know = 99, if > 98 enter 98)*

SIS

172. How many sisters do you have, living and dead?

|__|__| *number of sisters*

00	*None*		*Go to question 181*
88	*Refused*	*(DO NOT READ)*	*Go to question 181*
99	*Don't know/Not sure*	*(DO NOT READ)*	*Go to question 181*

SISCRC

If the respondent has more than 1 sister ask:

173. How many of your sisters have ever had bowel cancer?

If respondent has told you that their sister is alive, ask:
Has your sister ever had bowel cancer?

If respondent has told you that their sister has died, ask:
Did you sister ever have bowel cancer?

|__|__| *number of sisters*

00	*None*	*Go to question 175*
88	*Refused*	*Go to question 175*
99	*Don't know/Not sure*	*Go to question 175*

SISCRCDX

174. How old [*were they/was she*] when it was first diagnosed?

Probe for age at diagnosis for all sisters who had bowel cancer.

|__|__| *years old (Don't know = 99, if > 98 enter 98)*

|__|__| *years old (Don't know = 99, if > 98 enter 98)*

|__|__| *years old (Don't know = 99, if > 98 enter 98)*

|__|__| *years old (Don't know = 99, if > 98 enter 98)*

|__|__| *years old (Don't know = 99, if > 98 enter 98)*

SISBC

If the respondent has more than 1 sister ask:

175. How many of your sisters have ever had breast cancer?

If respondent has told you that their sister is alive, ask:
Has your sister ever had breast cancer?

If respondent has told you that their sister has died, ask:
Did you sister ever have breast cancer?

|__|__| *number of sisters*

00 None	*Go to question 177*
88 Refused	*Go to question 177*
99 Don't know/Not sure	*Go to question 177*

SISBCDX

176. How old [*were they/was she*] when it was first diagnosed?

Probe for age at diagnosis for all sisters who had breast cancer.

|__|__| *years old (Don't know = 99, if > 98 enter 98)*

|__|__| *years old (Don't know = 99, if > 98 enter 98)*

|__|__| *years old (Don't know = 99, if > 98 enter 98)*

|__|__| *years old (Don't know = 99, if > 98 enter 98)*

|__|__| *years old (Don't know = 99, if > 98 enter 98)*

SISHA

[If the respondent has more than 1 sister ask:

177. How many of your sisters have ever had a '<u>heart attack</u>'? (also called a myocardial infarction)

If respondent has told you that their sister is alive, ask:
Has your sister ever had a heart attack? (also called a myocardial infarction)

If respondent has told you that their sister has died, ask:
Did your sister ever have a heart attack? (also called a myocardial infarction)

|__|__| *number of sisters*

00 None *Go to question 179*

88 Refused *Go to question 179*

99 Don't know/Not sure *Go to question 179*

SISHADX

If the respondent has any sisters alive ask:

178. How old were they when they had their first heart attack?

If respondent has told you that their sister has died, ask:
How old was she when she had her first heart attack?

Probe for age at time of first heart attack for all sisters who had a heart attack.

|__|__| *years old (Don't know = 99, if > 98 enter 98)*

|__|__| *years old (Don't know = 99, if > 98 enter 98)*

|__|__| *years old (Don't know = 99, if > 98 enter 98)*

|__|__| *years old (Don't know = 99, if > 98 enter 98)*

|__|__| *years old (Don't know = 99, if > 98 enter 98)*

SISSTK

If the respondent has more than 1 sister ask:

179. How many of your sisters have ever had a stroke?

If the respondent has only 1 sister who is alive ask:

Has your sister ever had a stroke?

If respondent has told you that their sister has died, ask:

Did your sister ever have a stroke?

|__|__| *number of sisters*

00 None		*Go to question 181*
88 Refused	*(DO NOT READ)*	*Go to question 181*
99 Don't know/Not sure	*(DO NOT READ)*	*Go to question 181*

SISSTKDX

If the respondent has more than 1 sister ask:

180. How old were they when they had their first stroke?

[Else ask:

How old was she when she had her first stroke?

Probe for age at time of first stroke for all sisters who had a stroke.

|__|__| *years old (Don't know = 99, if > 98 enter 98)*

|__|__| *years old (Don't know = 99, if > 98 enter 98)*

|__|__| *years old (Don't know = 99, if > 98 enter 98)*

|__|__| *years old (Don't know = 99, if > 98 enter 98)*

|__|__| *years old (Don't know = 99, if > 98 enter 98)*

BRO

181. How many brothers do you have, living and dead?

|__|__| *number of brothers*

00 None		*Go to NEXT SECTION*

[If participant is MALE go to question 190, if participant if FEMALE go to question 195]

88 Refused	*(DO NOT READ)*	*Go to NEXT SECTION*

[If participant is MALE go to question 190, if participant if FEMALE go to question 195]

99 Don't know/Not sure	*(DO NOT READ)*	*Go to NEXT SECTION*

[If participant is MALE go to question 190, if participant if FEMALE go to question 195]

BROCRC

[If the respondent has more than 1 brother ask:

182. How many of your brothers have ever had bowel cancer?

If the respondent has only 1 brother who is alive ask:

Has your brother ever had bowel cancer?

If respondent has told you that their brother has died, ask:

Did your brother ever have bowel cancer?

|__|__| *number of brothers*

00 None		*Go to question 184*
88 Refused	*(DO NOT READ)*	*Go to question 184*
99 Don't know/Not sure	*(DO NOT READ)*	*Go to question 184*

BROCRCDX

183. How old [*were they/was he*] when it was first diagnosed?

Probe for age at diagnosis for all brothers who had bowel cancer.

|__|__| *years old (Don't know = 99, if > 98 enter 98)*

|__|__| *years old (Don't know = 99, if > 98 enter 98)*

|__|__| *years old (Don't know = 99, if > 98 enter 98)*

|__|__| *years old (Don't know = 99, if > 98 enter 98)*

|__|__| *years old (Don't know = 99, if > 98 enter 98)*

BROPC

If the respondent has more than 1 brother ask:

184. How many of your brothers have ever had prostate cancer?

If the respondent has only 1 brother who is alive ask:
Has your brother ever had prostate cancer?

If respondent has told you that their brother has died, ask:
Did your brother ever have prostate cancer?

|__|__| *number of brothers*

00 None		*Go to question 186*
88 Refused	*(DO NOT READ)*	*Go to question 186*
99 Don't know/Not sure	*(DO NOT READ)*	*Go to question 186*

BROPCDX

185. How old [*were they/was he*] when it was first diagnosed?

Probe for age at diagnosis for all brothers who had prostate cancer.

|__|__| *years old (Don't know = 99, if > 98 enter 98)*

|__|__| *years old (Don't know = 99, if > 98 enter 98)*

|__|__| *years old (Don't know = 99, if > 98 enter 98)*

|__|__| *years old (Don't know = 99, if > 98 enter 98)*

|__|__| *years old (Don't know = 99, if > 98 enter 98)*

BROHA

If the respondent has more than 1 brother ask:

186. How many of your brothers have ever had a heart attack? (also called a myocardial infarction)

[If the respondent has only 1 brother who is alive ask:
Has your brother ever had a heart attack? (also called a myocardial infarction)

If respondent has told you that their brother has died, ask:
Did your brother ever have a heart attack? (also called a myocardial infarction)

|__|__| *number of brothers*

00 None		*Go to question 188*
88 Refused	*(DO NOT READ)*	*Go to question 188*
99 Don't know/Not sure	*(DO NOT READ)*	*Go to question 188*

BROHADX

If the respondent has more than 1 brother ask:

187. How old were they when they had their first heart attack?

[Else ask:

How old was he when he had his first heart attack?

Probe for age at time of first heart attack for all brothers who had heart attack.

|__|__| *years old (Don't know = 99, if > 98 enter 98)*

|__|__| *years old (Don't know = 99, if > 98 enter 98)*

|__|__| *years old (Don't know = 99, if > 98 enter 98)*

|__|__| *years old (Don't know = 99, if > 98 enter 98)*

|__|__| *years old (Don't know = 99, if > 98 enter 98)*

BROSTK

If the respondent has more than 1 brother ask:

188. How many of your brothers have ever had a stroke?

If the respondent has only 1 brother who is alive ask:

Has your brother ever had a stroke?

If respondent has told you that their brother has died, ask:

Did your brother ever have a stroke?

|__|__| *number of brothers*

00 None *Go to NEXT SECTION*

[If participant is MALE go to question 190, if participant if FEMALE go to question 195]

88 Refused *(DO NOT READ)* *Go to NEXT SECTION*

[If participant is MALE go to question 190, if participant if FEMALE go to question 195]

99 Don't know/Not sure *(DO NOT READ)* *Go to NEXT SECTION*

[If participant is MALE go to question 190, if participant if FEMALE go to question 195]

BROSTKDX

If the respondent has more than 1 brother ask:

189. How old were they when they had their first stroke?

[Else ask:

How old was he when he had his first stroke?

Probe for age at time of first stroke for all brothers who had strokes.

|__|__| *years old (Don't know = 99, if > 98 enter 98)*

|__|__| *years old (Don't know = 99, if > 98 enter 98)*

|__|__| *years old (Don't know = 99, if > 98 enter 98)*

|__|__| *years old (Don't know = 99, if > 98 enter 98)*

|__|__| *years old (Don't know = 99, if > 98 enter 98)*

SECTION 10 QUESTIONS FOR MEN ONLY

The next questions are about men's health.

BPH

190. The next questions are about prostate problems. As some men get older, they develop an enlarged prostate, which is known as benign prostatic hypertrophy or BPH.

Has a doctor ever told you that you have an enlarged prostate?

1	*Yes*	
2	*No*	*Go to question 192*
8	*Refused*	*Go to question 192*
9	*Don't know/Not sure*	*Go to question 192*

BPHUNT

191. When did the doctor first tell you that you have an enlarged prostate?

BPHAGE/BPHYR/BPHYRS/BPHMTHS

INTERVIEWER INSTRUCTION:
If year is current year probe for months ago.

Age |__|__| or Year |__|__||__|__| or Years ago |__|__| or Months ago |__|__|

[Refused, age = 88, Don't know, age = 99]

PSA

192. The next questions are about PSA tests for prostate cancer.

The PSA test, also known as the prostate specific antigen test, is the most common test for prostate cancer.

For this test, the doctor takes a small sample of blood and sends it to a laboratory for analysis.

Have you ever had a PSA test?

1	*Yes*		
2	*No*		*Go to NEXT SECTION – 'Drugs'*
8	*Refused*	*(DO NOT READ)*	*Go to NEXT SECTION – 'Drugs'*
9	*Don't know/Not sure*	*(DO NOT READ)*	*Go to NEXT SECTION – 'Drugs'*

PSALST

193. When was your LAST PSA test?

PSAAGE2/PSAYR2/PSAYRS2/PSAMTHS2

INTERVIEWER INSTRUCTION:
If year is current year probe for months ago.

Age |__|__| or Year |__|__||__|__| or Years ago |__|__| or Months ago |__|__|

[Refused, age = 88, Don't know, age = 99]

PSAFRST

194. When was your FIRST PSA test?

PSAAGE/PSAYR/PSAYRS/PSAMTHS

INTERVIEWER INSTRUCTION:
If year is current year probe for months ago.

Age |__|__| or Year |__|__||__|__| or Years ago |__|__| or Months ago |__|__|

[Refused, age = 88, Don't know, age = 99]

SECTION 11 QUESTIONS FOR WOMEN ONLY

The next questions are about women's health.

INTERVIEWER INSTRUCTION:
If participant is 65 years of age or older skip to question 216.

PILLPAST

195. The next question is about your use of birth control pills. Birth control pills are also known as oral contraceptives.

Since *{year of last contact}* **have you used birth control pills at all?**

1	*Yes*		
2	*No*		*Go to question 198*
8	*Refused*	*(DO NOT READ)*	*Go to question 198*
9	*Don't know/Not sure*	*(DO NOT READ)*	*Go to question 198*

PILLNOW

196. Are you currently using birth control pills?

1	*Yes*		*Go to question 198*
2	*No*		
8	*Refused*	*(DO NOT READ)*	*Go to question 198*
9	*Don't know/Not sure*	*(DO NOT READ)*	*Go to question 198*

PILLSTOP

197. When did you last stop using birth control pills?

PILLAGE/PILLYR/PILLYRS/PILLMTHS

INTERVIEWER INSTRUCTION:

If year is current year probe for months ago.

Age |__|__| or Year |__|__||__|__| or Years ago |__|__| or Months ago |__|__|

[Refused, age = 88, Don't know, age = 99]

HYST

198. Now I will ask you some questions about having a hysterectomy.

INTERVIEWER CLARIFICATION: A hysterectomy is an operation in which the womb or uterus is removed. One or both ovaries are often removed at the same time

Have you had a hysterectomy?

1	*Yes*		
2	*No*		*Go to question 200*
8	*Refused*	*(DO NOT READ)*	*Go to question 200*

9 Don’t know/Not sure (DO NOT READ) Go to question 200

HYSTUNT

199. When did you have your <u>hysterectomy</u>?

HYSTAGE/HYSTYR/HYSTYRS/HYSTMTHS

INTERVIEWER INSTRUCTION:
If year is current year probe for months ago.

Age |__|__| or Year |__|__||__|__| or Years ago |__|__| or Months ago |__|__|

[Refused, age = 88, Don’t know, age = 99]

OVA

200. Have you had one or both of your ovaries removed?

1 Yes

2 No Go to question 203

8 Refused (DO NOT READ) Go to question 203

9 Don’t know/Not sure (DO NOT READ) Go to question 203

OVANUM

201. How many ovaries were removed?

1 One

2 Two

8 Refused (DO NOT READ)

9 Don’t know/Not sure (DO NOT READ)

OVAUNT

202. When was/were your ovary/ies removed?

OVAAGE/OVAYR/OVAYRS/OVAMTHS

INTERVIEWER INSTRUCTION:
If year is current year probe for months ago.

Age |__|__| or Year |__|__||__|__| or Years ago |__|__| or Months ago |__|__|

[If at time of hysterectomy, enter age = 90]

[Refused, age = 88, Don’t know, age = 99]

PRDS

203. Have you had a menstrual period in the last 12 months?

[If a woman tells you that they stopped, but then started again after she started taking HRT, enter the response as 3 “stopped, then started again after starting HRT” and ask Question 205]

1	*Yes*		*Go to question 207*
2	*No*		
3	*Yes, stopped, then started again after starting HRT*		*Go to question 205*
8	*Refused*	*(DO NOT READ)*	*Go to question 207*
9	*Don't know/ Not sure*	*(DO NOT READ)*	*Go to question 207*

PRDSTP

204. When was your last menstrual period?

PRDAGE/PRDYR/PRDYRS/PRDMTHS

Age |__|__| or Year |__|__||__|__| or Years ago |__|__| or Months ago |__|__|

Go to question 206

[If at time of hysterectomy or in the month before hysterectomy, enter age = 90 & GO TO question 207]

[If at time ovaries were removed, or in the month before ovaries removed, enter age = 91 & GO TO question 207]

[refused, age = 88; don't know, age = 99]

PRDSTP2

205. When did your menstrual periods first stop?

PRDAGE2/PRDYR2/PRDYRS2/PRDMTHS2

[INTERVIEWER CLARIFICATION: for women who told you that their periods stopped, then started again after they started on HRT, this refers to when they first stopped]

Age |__|__| or Year |__|__||__|__| or Years ago |__|__| or Months ago |__|__|

[If at time of hysterectomy or in the month before hysterectomy, enter age = 90 & GO TO question 207]

[If at time ovaries were removed, or in the month before ovaries removed, enter age = 91 & GO TO question 207]

[refused, age = 88; don't know, age = 99]

PRDSRESN

206. Why did your menstrual periods stop?

READ OUT

1 Natural menopause

2 Hysterectomy

3 Had ovaries removed

4 Other, specify ________________

8 Refused (DO NOT READ)

9 Don't know/Not sure (DO NOT READ)

HRT

207. The next questions are about use of hormone replacement therapy, which is also known as HRT.

HRT is medication prescribed by a doctor, such as oestrogen, progesterone or other female hormones.

INTERVIEWER CLARIFICATION: HRT might be pills, patches, implants, injections, vaginal creams or vaginal suppositories.

Have you ever used any HRT for more than 1 month?

1 Yes

2 No — Go to question 212

8 Refused (DO NOT READ) — Go to question 212

9 Don't know/Not sure (DO NOT READ) — Go to question 212

HRTSTR

208. When did you start using HRT?

HRTAGE1/HRTYR1/HRTYRS1/HRTMTHS1

INTERVIEWER INSTRUCTION:
If year is current year probe for months ago.

Age |__|__| or Year |__|__||__|__| or Years ago |__|__| or Months ago |__|__|

[Refused, age = 88, Don't know, age = 99]

HRTPRDS

209. Were you still having periods when you first took HRT?

1 Yes

2 No

8 Refused (DO NOT READ)

9 Don't know/Not sure (DO NOT READ)

HRTNOW

210. Are you currently taking HRT?

1 Yes — Go to question 212

2 No

8 Refused (DO NOT READ) — Go to question 212

9 Don't know/Not sure (DO NOT READ) — Go to question 212

HRTSTP

211. When did you last stop taking HRT?

HRTAGE2/HRTYR2/HRTYRS2/HRTMTHS2

INTERVIEWER INSTRUCTION:
If year is current year probe for months ago.

Age |__|__| or Year |__|__||__|__| or Years ago |__|__| or Months ago |__|__|

[Refused, age = 88, Don't know, age = 99]

AHRT

212. Have you used any over-the-counter, alternative or complementary preparations for hormone replacement or to treat symptoms of menopause? These preparations include herbal, natural or soy-based products but do not include foods.

1	*Yes*		
2	*No*		*Go to question 216*
8	*Refused*	*(DO NOT READ)*	*Go to question 216*
9	*Don't know/Not sure*	*(DO NOT READ)*	*Go to question 216*

AHRTSTR

213. When did you start using over-the-counter preparations for hormone replacement?

AHRTAGE1/AGEHRTYR1/AHRTYRS1/AHRTMTH1

INTERVIEWER INSTRUCTION:
If year is current year probe for months ago.

Age |__|__| or Year |__|__||__|__| or Years ago |__|__| or Months ago |__|__|

[Refused, age = 88, Don't know, age = 99]

AHRTNOW

214. Are you currently taking over-the-counter preparations for hormone replacement?

1	*Yes*		*Go to question 216*
2	*No*		
8	*Refused*	*(DO NOT READ)*	*Go to question 216*
9	*Don't know/Not sure*	*(DO NOT READ)*	*Go to question 216*

AHRTSTP

215. When did you last stop taking over-the-counter preparations for hormone replacement?

AHRTAGE2/AHRTYR2/AHRTYRS2/AHRTMTH2

INTERVIEWER INSTRUCTION:
If year is current year probe for months ago.

Age |__|__| or Year |__|__||__|__| or Years ago |__|__| or Months ago |__|__|

[Refused, age = 88, Don't know, age = 99]

MAMM

216. The next questions are about mammograms.

INTERVIEWER CLARIFICATION: A mammogram is a breast x-ray that is used to identify abnormalities in the breast. It can detect breast cancer when there are no symptoms.

Have you ever had a mammogram?

1	*Yes*		
2	*No*		*Go to NEXT SECTION - Drugs*
8	*Refused*	*(DO NOT READ)*	*Go to NEXT SECTION - Drugs*
9	*Don't know/Not sure*	*(DO NOT READ)*	*Go to NEXT SECTION - Drugs*

MAMMLST

217. When was your last mammogram?

MAMMAGE/MAMMYR/MAMMYRS/MAMMMTHS

INTERVIEWER INSTRUCTION:
If year is current year probe for months ago.

Age |__|__| or Year |__|__||__|__| or Years ago |__|__| or Months ago |__|__|

[Refused, age = 88, Don't know, age = 99]

SECTION 12 DRUGS

INTERVIEWER INSTRUCTION:
Only conduct drug audit for 'Telephone Interview before Clinical assessment'.

Else ask only questions 223 to 225 (inclusive).

DRG

INTERVIEWER INSTRUCTION
In this section, unlike in the rest of the questionnaire, do not probe "don't know/not sure" responses. Thus, if the person responds, "don't know", record it and move immediately to the next question.

218. Now I will ask about medicines that you take regularly. These might be medicines prescribed by your doctor for such things as blood pressure or arthritis, or medicines you buy over the counter. These do not include vitamins and other dietary supplements you might take.

Do you usually take any prescription or over the counter medicines at least once per week?

1 Yes

2 No *Go to question 223*

8 Refused *(DO NOT READ) Go to NEXT SECTION – "End of Interview"*

9 Don't Know/Not Sure *(DO NOT READ) Go to NEXT SECTION – "End of Interview"*

DRGWTH

219. Do you have these medications with you?

1 Yes

2 No *Go to question 221*

DRGABLE

INTERVIEWER INSTRUCTION
If you are in doubt as to whether something should be classified as a dietary supplement or a medicine, record it as a medicine.

220. Good, can you read the medication names out to me please?

1 Yes *Complete the DRUG AUDIT form*

2 No

DRGKNOW

221. Do you know the names of the medications that you take?

1 Yes

2 No *Go to question 226*

DRUG AUDIT FORM - Record drug details here. Attach additional sheets if necessary

Drug name	Strength (eg 200 mg)	Dose (eg 1, 2)	Frequency (Record as written on the box)	Duration (Use codes below)

Once complete - Go to next page

Duration codes

1	< 1 year
2	1-2 years

3	3-4 years
4	5-9 years

5	10+ years
8	Not asked

9	Don’t know

ASPASK

INTERVIEWER QUESTION [DO NOT READ OUT]:

222. *Is aspirin listed on the drug audit?*

[Some common brand names of aspirin are Aspro, Cardiprin, Cartia, Disprin and Solprin]

1	*Yes*		*Go to NEXT SECTION – question 244*
2	*No*		

ASP

223. The next questions are about aspirin. Some common brand names of aspirin are Aspro, Cardiprin, Cartia, Disprin and Solprin.

Do you usually take aspirin at least once per week?

1	*Yes*		
2	*No*		*Go to NEXT SECTION – question 244*
8	*Refused*	*(DO NOT READ)*	*Go to NEXT SECTION – question 244*
9	*Don't Know/Not Sure*	*(DO NOT READ)*	*Go to NEXT SECTION – question 244*

ASPUNT

224. How often do you usually take aspirin?

1	*1 day per week*	
2	*2-3 days per week*	
3	*4-6 days per week*	
4	*7 days per week*	
8	*Refused*	*(DO NOT READ)*
9	*Don't Know/Not Sure*	*(DO NOT READ)*

ASPTIM

225. For how many years have you taken aspirin at least once per week?

1	*Less than one year*	
2	*1 to 2 years*	
3	*2 to 4 years*	
4	*5-9 years*	
5	*10 or more years*	
8	*Refused*	*(DO NOT READ)*
9	*Don't Know/Not Sure*	*(DO NOT READ)*

INTERVIEWER INSTRUCTION:
END OF QUESTIONNAIRE for all participants except those that did not have their medications or cannot remember the names of their medications.

GO TO NEXT SECTION – question 244, 'End of Interview'

DRGBP

The remaining questions in this section are asked of people who did not have their medications and those who do not know the names of their medications.

226. Do you take any medication prescribed by a doctor to control your blood pressure?

1 Yes

2 No

8 Refused (DO NOT READ)

9 Don't Know/Not Sure (DO NOT READ)

DRGCHOL

227. Do you take any medication prescribed by a doctor to lower your cholesterol?

1 Yes

2 No

8 Refused (DO NOT READ)

9 Don't Know/Not Sure (DO NOT READ)

DRGBLOD

228. Do you take warfarin to thin your blood?

1 Yes

2 No

8 Refused (DO NOT READ)

9 Don't Know/Not Sure (DO NOT READ)

DRGANG

229. Do you take any medication prescribed by a doctor for angina?

1 Yes

2 No

8 Refused (DO NOT READ)

9 Don't Know/Not Sure (DO NOT READ)

DIABINS

230. Do you inject insulin for diabetes (high blood sugar)?

1 Yes

2 No

8 *Refused* *(DO NOT READ)*

9 *Don't Know/Not Sure* *(DO NOT READ)*

DIABTAB

231. Do you take tablets prescribed by a doctor for <u>diabetes</u> (high blood sugar)?

1 *Yes*

2 *No*

8 *Refused* *(DO NOT READ)*

9 *Don't Know/Not Sure* *(DO NOT READ)*

ASP

232. The next questions are about aspirin. Some common brand names of aspirin are Aspro, Cardiprin, Cartia, Disprin and Solprin.

Do you usually take aspirin at least once per week?

1 *Yes*

2 *No* *Go to question 235*

8 *Refused* *(DO NOT READ)* *Go to question 235*

9 *Don't Know/Not Sure* *(DO NOT READ)* *Go to question 235*

ASPUNT

233. How often do you usually take aspirin?

1 *1 day per week*

2 *2-3 days per week*

3 *4-6 days per week*

4 *7 days per week*

8 *Refused* *(DO NOT READ)*

9 *Don't Know/Not Sure* *(DO NOT READ)*

ASPTIM

234. For how many years have you taken aspirin at least once per week?

1 *Less than one year*

2 *1 to 2 years*

3 *2 to 4 years*

4 *5-9 years*

5 *10 or more years*

8 *Refused* *(DO NOT READ)*

9 *Don't Know/Not Sure* *(DO NOT READ)*

NSAID

235. The next questions are about medicines that are usually called anti-inflammatories, or non-steroidal anti-inflammatories, which is often abbreviated to NSAIDs. Sometimes they are called strong painkillers.

Pronounce NSAIDS as N.S. AIDs (ie use the initials N and S, but pronounce the rest as "aids")

Celebrex, Mobic and Vioxx are three anti-inflammatory prescription medicines used to treat arthritis. Do you take any of them?

1	*Yes*		
2	*No*		*Go to question 238*
8	*Refused*	*(DO NOT READ)*	*Go to question 238*
9	*Don't Know/Not Sure*	*(DO NOT READ)*	*Go to question 238*

NSAIDUNT

236. How often do you take these medicines?

1	*1 day per week or less*		*Go to question 238*
2	*2-3 days per week*		
3	*4-6 days per week*		
4	*7 days per week*		
8	*Refused*	*(DO NOT READ)*	
9	*Don't Know/Not Sure*	*(DO NOT READ)*	

NSAIDTIM

237. For how many years have you taken these medicines?

1	*Less than one year*	
2	*1 to 2 years*	
3	*2 to 4 years*	
4	*5-9 years*	
5	*10 or more years*	
8	*Refused*	*(DO NOT READ)*
9	*Don't Know/Not Sure*	*(DO NOT READ)*

ANTIS

238. Do you take any other anti-inflammatories or strong painkilling medicines prescribed by a doctor? Some commonly prescribed ones are Voltaren, Brufen, Indocid, Naprosyn, Fenac, Feldene, Orudis and Oruvail. Please do not include aspirin, Panamax or Panadeine Forte or Paracetamol.

1	*Yes*		
2	*No*		*Go to question 241*
8	*Refused*	*(DO NOT READ)*	*Go to question 241*
9	*Don't Know/Not Sure*	*(DO NOT READ)*	*Go to question 241*

ANTISUNT

239. How often do you take these medicines?

1	*1 day per week or less*		*Go to question 241*
2	*2-3 days per week*		
3	*4-6 days per week*		
4	*7 days per week*		
8	*Refused*	*(DO NOT READ)*	
9	*Don't Know/Not Sure*	*(DO NOT READ)*	

ANTISTIM

240. For how many years have you taken these medicines?

1	*Less than one year*	
2	*1 to 2 years*	
3	*2 to 4 years*	
4	*5-9 years*	
5	*10 or more years*	
8	*Refused*	*(DO NOT READ)*
9	*Don't Know/Not Sure*	*(DO NOT READ)*

ANTI

241. Do you take any <u>over the counter</u> anti-inflammatories or strong painkillers at least once per week? Some common ones are Voltaren Rapid, Ibuprofen and Nurofen. Please do not include aspirin, paracetamol (which is the drug in Panadol, Panadeine, Dymadon or Tylenol) or any creams or ointments you may use.

1	*Yes*		
2	*No*		*Go to NEXT SECTION – question 244*
8	*Refused*	*(DO NOT READ)*	*Go to NEXT SECTION – question 244*
9	*Don't Know/Not Sure*	*(DO NOT READ)*	*Go to NEXT SECTION – question 244*

ANTIUNT

242. How often do you take these medicines?

- *1 1 day per week or less* *Go to NEXT SECTION – question 244*
- *2 2-3 days per week*
- *3 4-6 days per week*
- *4 7 days per week*
- *8 Refused (DO NOT READ)*
- *9 Don't Know/Not Sure (DO NOT READ)*

ANTITIM

243. For how many years have you taken these medicines at least once per week?

- *1 Less than one year*
- *2 1 to 2 years*
- *3 2 to 4 years*
- *4 5-9 years*
- *5 10 or more years*
- *8 Refused (DO NOT READ)*
- *9 Don't Know/Not Sure (DO NOT READ)*

SECTION 13 End of interview

ENDINT

244. Thank you, we have now finished the interview.

This concludes the Health 2020 Health and Lifestyle interview with

reference number____

of participant____

concluded on____

at ____

and conducted by ____

INTEND

INTERVIEWER INSTRUCTION:
Ask for 'Telephone Interview before Clinical Assessment' participants ONLY.

245. Thank you for taking the time to take part in this important interview today.

Forty one and a half thousand participants were recruited in the early 1990's. So far, we have follow up 24,000 of these participants. All of these participants have been able to provide a blood sample. It is important for us to collect a blood sample from all participants, including you.

There are two ways we can collect a blood sample, you can either visit a study centre close to you or a nurse can come to you. Which option would you prefer?

1 Visit a study centre

2 Home visit

8 Refused

SECTION 14 Interview quality

[After you have finished the interview please rate the quality of the interview according to the following questions.]

IQRESP

246. Was the subject responsive?

1 *not at all (uninterested, reticent)*
2 *fairly cooperative and responsive*
3 *very cooperative, responsive and interested*

IQSMK

247. In your opinion, how well did the respondent answer the questions on smoking?

1 *well*
2 *fairly well*
3 *not well*
8 *not applicable*

IQALC

248. In your opinion, how well did the respondent answer the questions on alcohol?

1 *well*
2 *fairly well*
3 *not well*
8 *not applicable*

IQPHYACT

249. In your opinion, how well did the respondent answer the questions on physical activity?

1 *well*
2 *fairly well*
3 *not well*
8 *not applicable*

IQHLTH

250. In your opinion, how well did the respondent answer the questions on his or her own health (illnesses and procedures)?

1 *well*
2 *fairly well*
3 *not well*
8 *not applicable*

IQFAMHX

251. In your opinion, how well did the respondent answer the questions on his or her family history of disease?

1 *well*
2 *fairly well*
3 *not well*
8 *not applicable*

IQMEN

252. In your opinion, how well did the respondent answer the questions on men's health?

1 *well*
2 *fairly well*
3 *not well*
8 *not applicable*

253. In your opinion, how well did the respondent answer the questions on women's health?

1 *well*
2 *fairly well*
3 *not well*
8 *not applicable*
