# Supplementary material for: Prospective Evaluation over 15 Years of Six Breast Cancer Risk Models
Source: Cancers (Basel). 2021 Oct 16;13(20):5194. doi: 10.3390/cancers13205194 (PMC8534072; doi:10.3390/cancers13205194)
Supplement: Supplementary file 1 [file cancers-13-05194-s001.zip › Supplementary_Material_20210929_final.pdf]

cancers

Supplementary Materials: Prospective evaluation over 15 years of six breast cancer risk models

Sherly X. Li, Roger L. Milne, Tú Nguyen-Dumont, Dallas R. English, Graham G. Giles, Melissa C. Southey, Antonis C. Antoniou, Andrew Lee, Ingrid Winship, John L. Hopper, Mary Beth Terry and Robert J. MacInnis

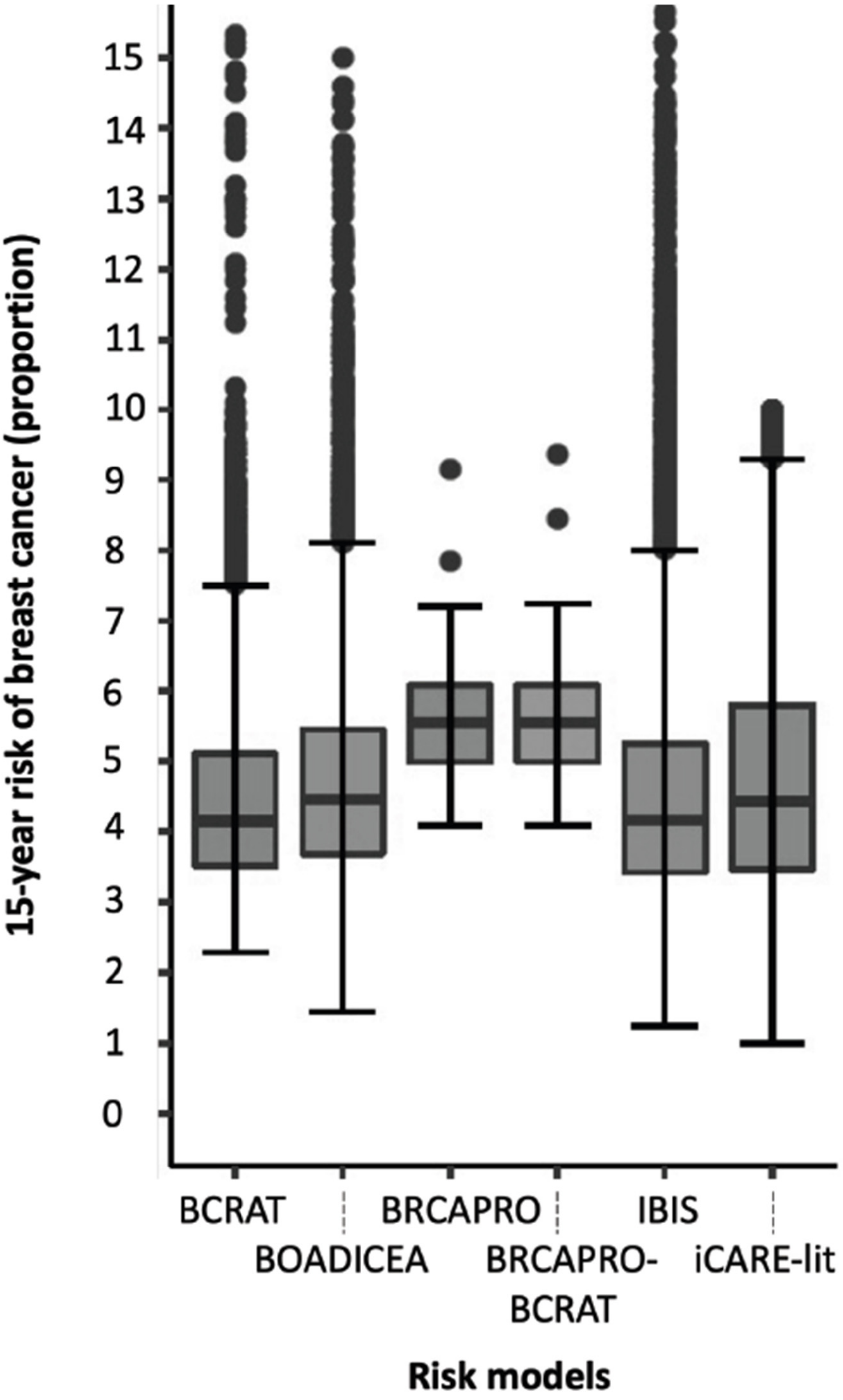

Figure S1. Boxplot of 15-year risk of breast cancer by risk prediction models.

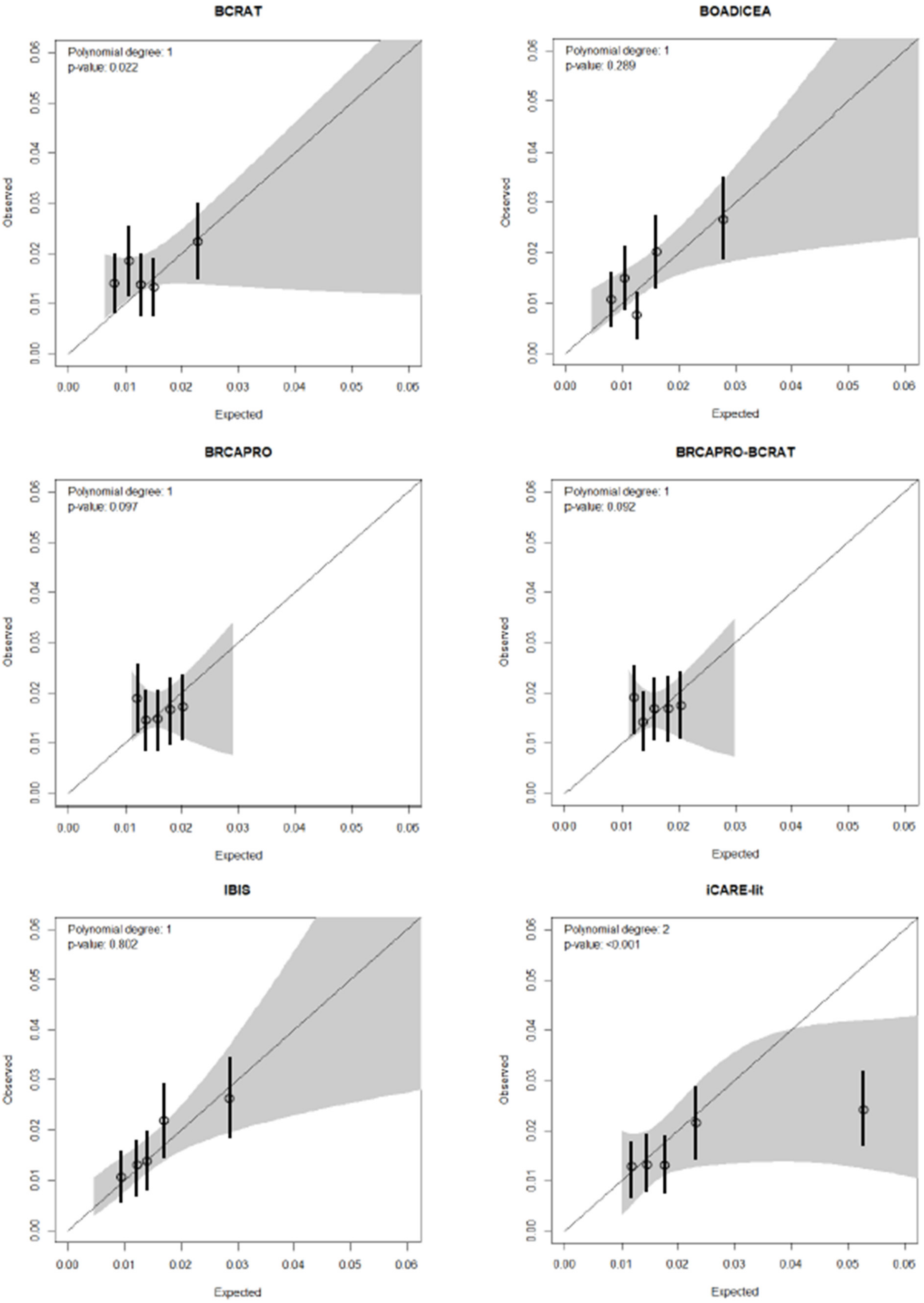

Figure S2. Calibration of 5-year breast cancer risk scores for six risk models across the risk spectrum.

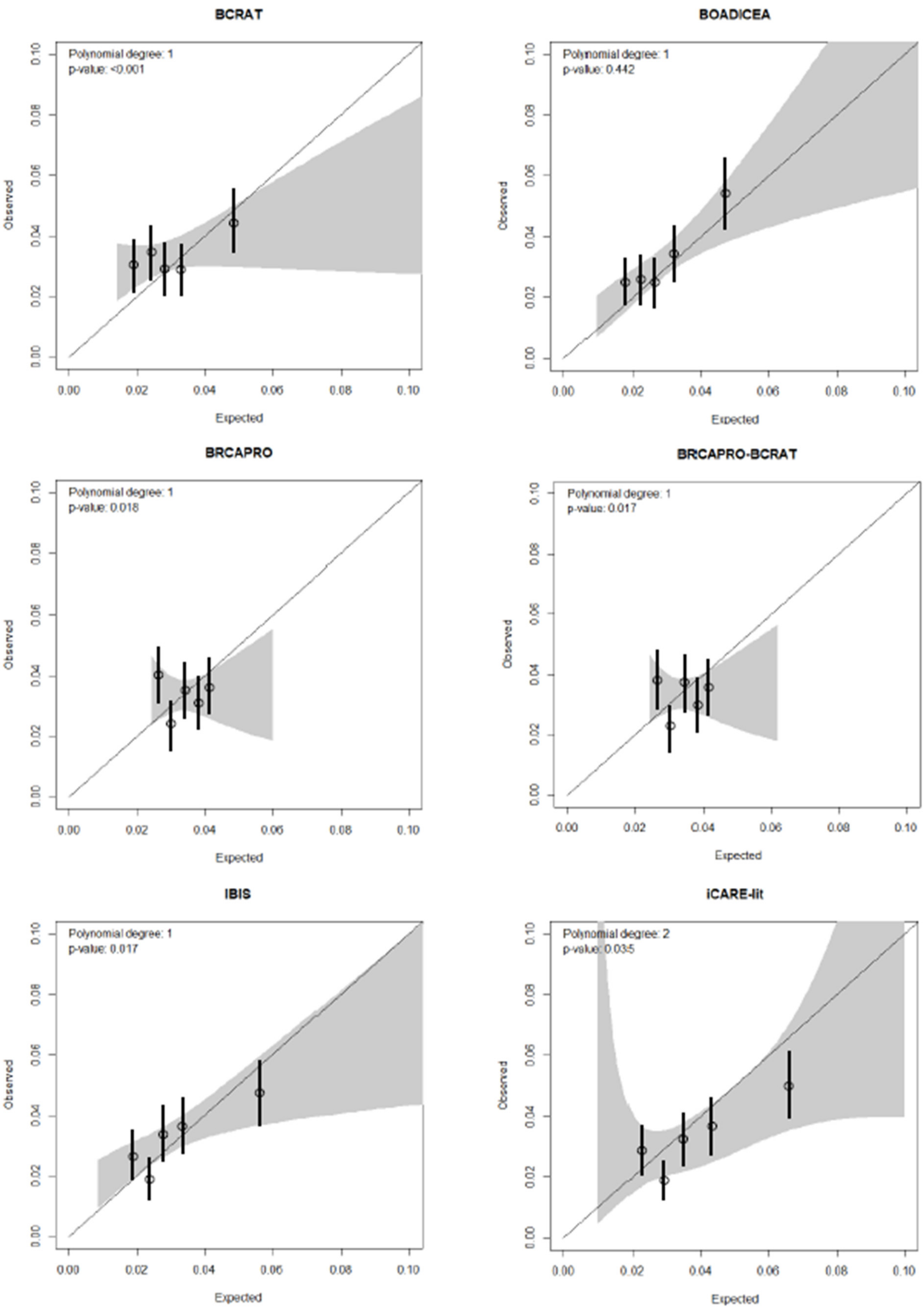

Figure S3. Calibration of 10-year breast cancer risk scores for six risk models across the risk spectrum.

Table S1. Summary of inputs for risk models and their availability within the Melbourne Collaborative Cohort Study.

Components	IBIS	BOADI-CEA	BRCAPRO	BRCAPRO-BCRAT	BCRAT	iCARE-lit	Availability in MCCS
Personal information							
Age	●	●	●	●	●	●	●
Year of birth		●					●
Ashkenazi Jewish heritage	●	●	●	●			
Ethnicity[1]	●	●	●	●	●	●	●
Height	●	●				●	●
Body mass index	●	●				●	●
Identical twin status		●	●	●			
Alcohol intake		●				●	●
Age at menarche	●	●		●	●	●	●
Number of live births	●	●				●	●
Age at first birth	●	●		●	●	●	●
Menopausal status	●	●				●	●
Age at menopause		●				●	●
Oral contraceptive pill use		●				●	●
Menopausal hormone therapy use	●	●				●	●
History of hyperplasia (atypical or typical)/ benign breast cancer	●			●	●	●	
History of lobular carcinoma in situ	●						●
Previous breast biopsy	●			●	●		
History of ovarian cancer	●	●					●
Mammographic density	●	●					*
BRCA1/*BRCA2* mutation status	●	●	●	●			*
ATM/*CHEK2*/*PALB2* mutation status		●					
Family history							
Breast cancer[2]	●	●	●	●	●	●	●
Contralateral breast cancer[3]	●	●	●	●			
Ovarian cancer	●	●	●	●			
Pancreatic cancer		●					
Prostate cancer		●					●
Age at cancer diagnosis	●	●	●	●			●
Underlying incidence rates	UK: 2005-2009 Thames Cancer Registry	Australia: 1982-2010 AIHW	US: 2014-2016 SEER (White)	US: 2014-2016 SEER (White)	US: White 1983-1987 SEER (White)	Australia: 2010-2015 AIHW (updated)	

* Not available for most participants eligible for this study, so these variables were not included in our analyses.

[1]IBIS and BOADICEA uses country-specific incidence rates of breast cancer, so they account for ethnicity indirectly.

[2]Breast cancer family history for IBIS and BOADICEA accounts for multigenerational history, including age of relative and diagnosis. BRCAPRO models accounts for multigenerational history, including relatives age of diagnosis and imputes missing ages. BCRAT includes number of first-degree relatives and iCARE includes a binary presence or absence of BC in first degree relatives.

[3]IBIS asks for bilateral breast cancer.

IBIS: International Breast Cancer Intervention Study model (IBIS or Tyrer-Cuzick version 8b); BOADICEA: the Breast and Ovarian Analysis of Disease Incidence and Carrier Estimation Algorithm model (version 5.0.0); BRCAPRO: BayesMendel (version 2.1-7); BRCAPRO-BCRAT (version 2.1-7); BCRAT: the Breast Cancer Risk Assessment Tool (version 4.1); iCARE-lit: Individualised Coherent Absolute Risk Estimators-literature model (version 1); MCCS: Melbourne Collaborative Cohort Study; SEER: the Surveillance, Epidemiology, and End Results; AIHW: Australian Institute of Health and Welfare.

Table S2. Sensitivity analysis of calibration and discrimination of 15-year risks for six risk models censoring in situ breast cancer[1].

Risk Model	Number of women	Expected number of cases	Observed number of cases	Expected/Observed ratio (95%CI)	Concordance statistic (95% CI)
Overall					
IBIS	7608	339.0	351	0.97 (0.87,1.07)	0.57 (0.52,0.62)
BOADICEA		340.8	351	0.97 (0.87,1.08)	0.61 (0.56,0.66)
BRCAPRO		387.5	351	1.10 (0.99,1.23)	0.53 (0.47,0.59)
BRCAPRO-BCRAT		387.8	351	1.10 (1.00,1.23)	0.52 (0.47,0.58)
BCRAT		326.3	351	0.93 (0.84,1.03)	0.52 (0.46,0.57)
iCARE-lit		338.0	351	0.96 (0.87,1.07)	0.53 (0.47,0.59)
Age 50-54 years	1912				
IBIS		89.5	91	0.98 (0.80,1.21)	0.59 (0.53,0.65)
BOADICEA		82.6	91	0.91 (0.74,1.11)	0.60 (0.54,0.66)
BRCAPRO		82.4	91	0.91 (0.74,1.11)	0.49 (0.43,0.55)
BRCAPRO-BCRAT		82.5	91	0.91 (0.74,1.11)	0.49 (0.43,0.55)
BCRAT		75.8	91	0.83 (0.68,1.02)	0.54 (0.48,0.60)
iCARE-lit		85.4	91	0.94 (0.76,1.15)	0.55 (0.49,0.62)
Age 55-59 years	2679				
IBIS		121.1	116	1.04 (0.87,1.25)	0.56 (0.50,0.61)
BOADICEA		123.5	116	1.06 (0.89,1.28)	0.59 (0.54,0.65)
BRCAPRO		134.2	116	1.16 (0.96,1.39)	0.54 (0.49,0.59)
BRCAPRO-BCRAT		134.3	116	1.16 (0.97,1.39)	0.54 (0.49,0.59)
BCRAT		114.1	116	0.98 (0.82,1.18)	0.58 (0.52,0.63)
iCARE-lit		119.7	116	1.03 (0.86,1.24)	0.51 (0.45,0.57)
Age 60-65 years	3017				
IBIS		128.5	144	0.89 (0.76,1.05)	0.58 (0.53,0.63)
BOADICEA		134.7	144	0.94 (0.79,1.10)	0.59 (0.54,0.64)
BRCAPRO		171.0	144	1.19 (1.01,1.40)	0.51 (0.46,0.56)
BRCAPRO-BCRAT		171.1	144	1.19 (1.01,1.40)	0.49 (0.44,0.54)
BCRAT		136.4	144	0.95 (0.80,1.12)	0.51 (0.46,0.56)
iCARE-lit		132.9	144	0.92 (0.78,1.09)	0.55 (0.50,0.60)
No family history of breast cancer[2]	5888				
IBIS		215.8	241	0.90 (0.79,1.02)	0.54 (0.50,0.58)
BOADICEA		239.9	241	1.00 (0.88,1.13)	0.56 (0.52,0.60)
BRCAPRO		299.1	241	1.24 (1.09,1.41)	0.50 (0.46,0.54)
BRCAPRO-BCRAT		299.3	241	1.24 (1.09,1.41)	0.50 (0.46,0.54)
BCRAT		228.7	241	0.95 (0.84,1.08)	0.53 (0.49,0.56)
iCARE-lit		254.1	241	1.05 (0.93,1.20)	0.52 (0.48,0.56)
Family history of breast cancer[2]	1720				
IBIS		123.1	110	1.12 (0.93,1.35)	0.57 (0.52,0.62)
BOADICEA		100.9	110	0.92 (0.76,1.11)	0.60 (0.55,0.65)
BRCAPRO		88.4	110	0.80 (0.67,0.97)	0.53 (0.47,0.59)
BRCAPRO-BCRAT		88.5	110	0.80 (0.67,0.97)	0.52 (0.47,0.58)
BCRAT		97.6	110	0.89 (0.74,1.07)	0.52 (0.46,0.57)
iCARE-lit		83.8	110	0.76 (0.63,0.92)	0.53 (0.47,0.59)

[1]Censor based on the earliest of censor date (1st March 2019, reaching 80 years of age, follow up time of 15 years or diagnosis of in situ breast cancer).

[2]Family history in first- or second-degree relatives

IBIS: International Breast Cancer Intervention Study model (IBIS or Tyrer-Cuzick version 8b); BOADICEA: the Breast and Ovarian Analysis of Disease Incidence and Carrier Estimation Algorithm model (version 5.0.0); BRCAPRO: BayesMendel (version 2.1-7);

BRCAPRO-BCRAT (version 2.1-7); BCRAT: the Breast Cancer Risk Assessment Tool (version 4.1); iCARE-lit: Individualised Coherent Absolute Risk Estimators-literature model (version 1)
